# Smart Multiplex Point-of-Care Platform for Simultaneous Drug Monitoring

**DOI:** 10.1021/acsami.3c06461

**Published:** 2023-07-27

**Authors:** Duygu Beduk, Tutku Beduk, José Ilton de Oliveira Filho, Abdellatif Ait Lahcen, Ebru Aldemir, Emine Guler Celik, Khaled Nabil Salama, Suna Timur

**Affiliations:** † Central Research Test and Analysis Laboratory Application and Research Center, Ege University, 35100 Bornova, Izmir, Turkey; ‡ Silicon Austria Labs (SAL) GmbH, Europastraße 12, 9500 Villach, Austria; § Sensors Lab, Advanced Membranes and Porous Materials Center, Computer, Electrical, and Mathematical Science and Engineering Division, King Abdullah University of Science and Technology (KAUST), Thuwal 23955-6900, Saudi Arabia; ∥ Department of Radiology, Weill Cornell Medicine, Dalio Institute for Cardiovascular Imaging, New York, New York 10021, United States; ⊥ Department of Psychiatry, Faculty of Medicine, Izmir Tinaztepe University, 35400 Buca, Izmir, Turkey; # Department of Bioengineering, Faculty of Engineering, Ege University, 35100 Bornova, Izmir, Turkey; ○ Department of Biochemistry, Faculty of Science, Ege University, 35100 Bornova, Izmir, Turkey

**Keywords:** multiplex sensing, laser-scribed graphene, illicit drugs, cocaine, amphetamine, benzodiazepine, point-of-care testing (POC)

## Abstract

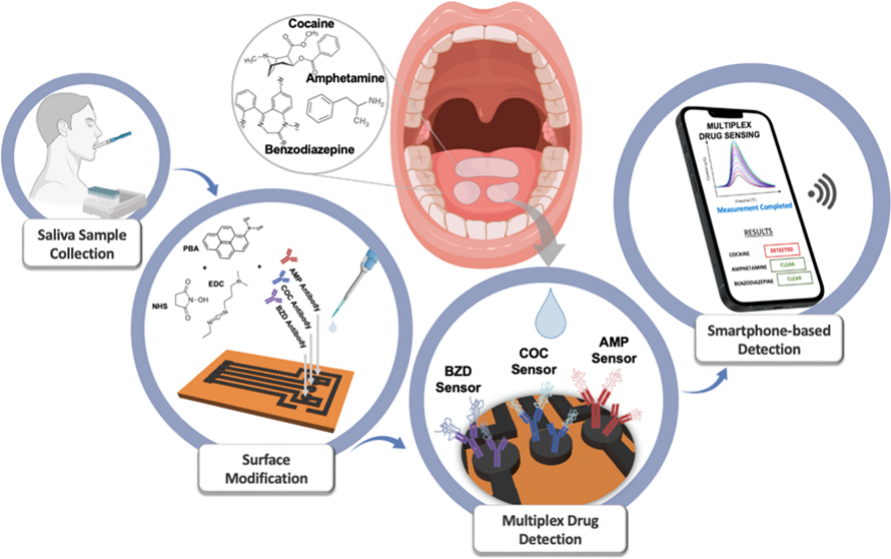

Recently, illicit drug use has become more widespread and is linked to problems with crime and public health. These drugs disrupt consciousness, affecting perceptions and feelings. Combining stimulants and depressants to suppress the effect of drugs has become the most common reason for drug overdose deaths. On-site platforms for illicit-drug detection have gained an important role in dealing, without any excess equipment, long process, and training, with drug abuse and drug trafficking. Consequently, the development of rapid, sensitive, noninvasive, and reliable multiplex drug-detecting platforms has become a major necessity. In this study, a multiplex laser-scribed graphene (LSG) sensing platform with one counter, one reference, and three working electrodes was developed for rapid and sensitive electrochemical detection of amphetamine (AMP), cocaine (COC), and benzodiazepine (BZD) simultaneously in saliva samples. The multidetection sensing system was combined with a custom-made potentiostat to achieve a complete point-of-care (POC) platform. Smartphone integration was achieved by a customized application to operate, display, and send data. To the best of our knowledge, this is the first multiplex LSG-based electrochemical platform designed for illicit-drug detection with a custom-made potentiostat device to build a complete POC platform. Each working electrode was optimized with standard solutions of AMP, COC, and BZD in the concentration range of 1.0 pg/mL–500 ng/mL. The detection limit of each illicit drug was calculated as 4.3 ng/mL for AMP, 9.7 ng/mL for BZD, and 9.0 ng/mL for COC. Healthy and MET (methamphetamine) patient saliva samples were used for the clinical study. The multiplex LSG sensor was able to detect target analytes in real saliva samples successfully. This multiplex detection device serves the role of a practical and affordable alternative to conventional drug-detection methods by combining multiple drug detections in one portable platform.

## Introduction

Drug abuse has detrimental impacts in terms of not only the mental and physical well-being of those who abuse drugs but also the social cost to the community.^1,2^ According to the United Nations Office on Drugs and Crime 2021 World Drug Report, almost 275 million people used drugs in the last year, with over 36 million people suffering from drug use problems. Recent worldwide statistics showed that over 5.5% of the population aged 15–64 years has used illegal drugs at least once in the last year, with 36.3 million people suffering from drug use problems. The number of people consuming drugs grew by 22% from 2010 to 2019, and the current estimation of global drug consumption is expected to increase by 11% by 2030.^3^

Drugs are chemical substances that can affect brain activity and exist in several forms, including powders, pills and capsules, liquids, and solids.^4^ They can be natural, semisynthetic, or entirely synthetic and are categorized as legal, illicit, or prescription-only.^5,6^ The drug type, purity, dosage, and route of consumption all have an impact on the human body.^7^ Drugs are classed as stimulants, hallucinogens, and depressants based on their physical and psychological impacts.^8^ Stimulants, often known as uppers, such as cannabis, cocaine, ecstasy, and amphetamine affect the central nervous system and make the user feel energized, focused, and alert.^1,9−11^ Depressants, also called downers, have a relaxing impact on the nervous system and anxiety, induce sleep, and reduce brain and nerve activity.^12^ Opioids (e.g., heroin, codeine, morphine, fentanyl), barbiturates (e.g., phenobarbital, thiopental), benzodiazepines (e.g., alprazolam, diazepam, clonazepam, lorazepam), and alcohol are classified as depressants.^13,14^ For medical conditions such as insomnia and obsessive-compulsive disorder, depressants are prescribed for treatment purposes.^9,15^ Abusing depressant use has become more widespread over time, especially when they are used with stimulants to counteract the depressive effects.^4^

Cocaine (COC) is a highly addictive stimulant extracted from coca leaves. It directly influences the neurological system minutes after consumption, provides euphoria, and relieves pain.^16^ COC is metabolized as benzoylecgonine, ecgonine, or ecgonine methyl ester, and can be found in biological fluids such as urine, blood, and saliva for up to three days after intake.^17^ The abuse leads to addiction, psychosis, insomnia, mental issues, and death.^18,19^ In Europe and the United States, COC use has gradually grown in recent years and is the second most often used banned substance.^17,20^ Amphetamine (AMP) is another addictive stimulant drug that can be used to make a variety of drugs and causes dopamine to be released resulting in an elevated mood and visual sensitivity. It has also been used to treat disorders such as narcolepsy.^21^

Benzodiazepines (BZDs) are used along with a stimulant like COC or AMP to counteract their stimulating effects. Consequently, increasing drug intake for more effectiveness of drugs increases the risk of overdosing.^15,22^ The growing consumption of these drugs has triggered the need for rapid, on-site, and sensitive drug detection in biofluids.^16,23^ Urine is considered the gold standard for noninvasive drug-detection matrices, but saliva, sweat, and hair have emerged as promising biological samples for drug detection.^24^ Saliva is a complex biofluid that has pharmacokinetic characteristics highly correlated with blood intoxication levels.^25^ It can be used for noninvasive on-site drug screening since it is simple to collect and can be easily used for the drug testing of drivers and workers.^26−28^ Because drugs have such a short half-life in the human body, it is challenging to detect their presence. Analytical detection methods such as high-performance liquid chromatography,^1^ gas chromatography,^29^ mass spectrometry,^30^ ion mobility spectrometry,^31^ Raman spectroscopy,^32^ Fourier transform infrared spectroscopy,^33^ nuclear magnetic resonance,^34^ and X-ray diffractometry^35^ accurately detect illegal drugs in the system. Despite their high accuracy and specificity, they suffer from a lack of accessibility and portability due to high cost, the need for trained personnel, a time-consuming and complex procedure, and the necessity for sample preparation.^15,17^ Miniaturization and affordability, including cost-effectiveness, portability, flexibility, ease of use without pretreatment, and high sensitivity, have accelerated the use of electrochemical-based biosensors for drug abuse detection in oral fluid.^36^ Highly compatible with on-site measurements, the electrochemical biosensors require a compact and portable design with the ability to perform, with a short response time, real-time illicit-drug detection in human samples.^18^ For this reason, the ability of customized miniaturized and portable electrochemical detection devices to provide high accessibility for both personal and in-field applications has been gaining significant attention in recent years.^37^ Another crucial requirement for illicit-drug detection is the necessity of simultaneous measurements of multiple drugs. Especially along with the COVID-19 pandemic, multiple drug abuse rates have gradually increased.^38^ For this reason, the identification of drug abuse in the human body requires specific attention.

In the current study, we designed a multiplex LSG-based sensing platform for the rapid and on-site detection of illicit drugs such as COC, AMP, and BZD in real saliva samples. Graphene has advantages such as the surface-to-volume ratio, high conductivity, stable carrier mobility, and exceptional electrical and mechanical properties compared to the other allotropes of carbon.^39−41^ These characteristics of graphene make it unique and highly sensitive for biosensing applications.^42−44^ LSGs have emerged rapidly as sensing platforms thanks to their advantages, such as high sensitivity, portability, low cost, and easy adaptability to a smartphone-based POC device.^45,46^ We have developed a multiplex homemade and portable potentiostat device operable with a custom-made smartphone application. Since using stimulants and depressants together became one of the most fatal drug abuse methods and increased rapidly, our sensing system aimed to detect the usage of stimulants and depressants at the same time with its multiplex structure and can detect all three drugs simultaneously in one saliva sample. This work can lead the way toward practical, smart, and innovative sensing platforms for illicit drugs. A clinical study was conducted with healthy and MET (methamphetamine) patient saliva samples.

The reliability and high sensitivity of the multiplex sensor was tested with spiked real samples. A multi-LSG sensor has been integrated into the potentiostat, sending electrochemical signals to the smartphone application, which makes this sensing system suitable for on-site detection such as workplace testing, borders, and roadside drug detection.

## Methods

### Methods and Apparatus

1-Pyrenebutyric acid (PBA), dimethyl sulfoxide (DMSO), 1-ethyl-3-(3-dimethyl aminopropyl) carbodiimide (EDC), *N*-hydroxysuccinimide (NHS), potassium chloride (KCl), bovine serum albumin (BSA, lyophilized powder, ≥96%), and codeine were purchased from Sigma-Aldrich (St. Louis, MO). Potassium ferrocyanide (K_4_[Fe(CN)_6_]) and ferricyanide (K_3_[Fe(CN)_6_]) were purchased from MP Biomedicals. The phosphate-buffered saline (PBS) with pH 7.4 and containing 0.0027 M potassium chloride (KCl) and 0.137 M sodium chloride (NaCl) was purchased from Fisher Bioreagents. coc-mAb1 was purchased from Artron BioResearch Inc. (cat.no. A10-Ab1). Benzoylecgonine-D8, amphetamine, benzodiazepine standard-3, and methamphetamine solutions were purchased from Cerilliant (Cerilliant Corp., Round Rock, TX). Benzodiazepine internal standard-3 solution was purchased from Sigma-Aldrich and consists of 7-aminoclonazepam-D4 (0.5 mg/mL), 7-aminoflunitrazepam-D7 (0.5 mg/mL), and α-hydroxytriazolam-D4 (1.0 mg/mL). Benzodiazepine mAb and amphetamine mAb were purchased from Arista Biologicals (cat.no. ABAMP-0400 and ABBZO-0400). Tetrahydrocannabinol (THC) was purchased from THC Pharm (Biochem. the-pharm, Germany). Pregabalin (Lyrica) was purchased from Pfizer Inc. Oral swab cotton pads (SOS) and swab storage tubes (STT) were purchased from Salimetrics LLC (Pennsylvania). Laser patterning was performed by a CO_2_ Universal Laser System (PLS6.75 laser). The commercial polyimide (PI) substrate (Kapton width: 12″) was purchased from Utech Products and used as the substrate. Electrochemical techniques, differential pulse voltammetry (DPV), and cyclic voltammetry (CV) were conducted by a PalmSens potentiostat instrument (Palm Instruments, Houten, Netherlands) and a custom-made multiplex potentiostat, named KAUSTat. Elemental analysis and morphological characterizations were performed by X-ray photoelectron spectroscopy (XPS) and scanning electron microscopy (SEM) instruments purchased from Thermo Scientific and Thermo Fisher Scientific as Apreo S LoVac models, respectively.

### Preparation of the Multiplex Electrochemical Sensor

The LSG sensor was prepared following the previously reported fabrication procedure.^47^ The multiplex system has three LSG working electrodes, one LSG counter electrode, and one LSG reference electrode. Each working area has 3 mm diameter. The sensing area has 2–1.5 cm dimensions. The connections of each electrode are designed to have 0.6 mm width and 2.8 cm length. Further details about fabrication are explained in the Supporting Information. The working electrode surfaces were modified individually to detect three illicit drugs, namely, BZD, AMP, and COC, from the same saliva sample. As the first step, 6.0 μL of 25 mM 1-pyrenebutyric acid (PBA) prepared in DMSO was incubated onto each working electrode for 1 h at 4 °C. The active area of each working electrode was washed with 0.05 M phosphate-buffered saline (PBS) to remove the excess unreacted PBA. Then, 6.0 μL of EDC/NHS (50:50 mM) mixture was drop cast onto each working area and incubated for 2 h at 4 °C. The electrode surfaces were rinsed with 0.1 M PBS and left at room temperature for 30 min to dry. When working electrode surfaces were completely dried, the graphene area between connections and electrodes was passivated by a nail polish layer and dried for 30 min. The passivation process was selected as the last step before the functionalization for complete removal and blockage of access binders on the electrode surface. As the final step, 6.0 μL of BZD antibody was immobilized on WE1, COC antibody immobilized on WE2, and AMP antibody immobilized on WE3 for 16 h incubation at 4 °C. Antibody concentrations for BZD, AMP, and COC were selected as 25, 25 μg/mL, and 200 ng/mL, respectively. Before the multiplex measurements, the electrochemical performance of antibodies was tested separately. LSG sensors having one reference, one counter, and one working electrode were prepared and functionalized with each antibody with the same preparation procedure. Different concentrations of BZD, AMP, and COC analytes were first tested to serve the role of a reference point for further measurements. All dilutions were carried out in 0.1 M PBS at pH 7.4. To achieve the necessary sensitivity and specificity, the electrode surface needs to be blocked, minimizing the nonspecific interactions onto functionalized surfaces.

Bovine serum albumin (BSA) is one of the commonly used blocking agents to block activated amine or epoxy functional ends. After incubation of biorecognition molecules (antibody), the electrode surface was washed with PBS and 6.0 μL of 0.1 mg/mL BSA in 0.1 M KCl solution was placed onto the working electrode surface to block the unwanted active area as described before and maintain the stability of the sensing performances in complex matrices. To test the sensor performance, 6.0 μL of the solution was immobilized with different concentrations of analytes on the functionalized working electrode surfaces and incubated for 30 min. Specific antibody–analyte interaction was detected by the DPV method with different current changes of each working electrode. The changes in oxidation current values correspond to the bonding of immobilized drug-specific antibodies and the different concentrations of drug analytes. All electrochemical measurements were performed 6 times at pH 7.4. The scan rate for DPV was 50 mV/s, and the potential range was between −0.60 and +0.40 V. The potential pulse was 0.05 V and time pulse was 0.1 s with 0.01 potential step. All electrochemical performance test measurements were performed using the KAUSTat potentiostat in 5.0 mM [Fe(CN)_6_]^3–/4–^ containing 0.1 M PBS and 0.1 M KCl.

### Preparation of Human Saliva Samples

The clinical research was approved by the Clinical Research Ethics Committee of Tinaztepe Buca Hospital (Decision number: 07). Saliva samples were taken using an oral swab pad and placed into the tube, which contains interbedded two separate parts.

First, an oral swab pad was placed in the mouth for 1 min, under the tongue. Then the oral swab pad was placed inside the tube, and sample tubes were centrifuged for 10 min to drain saliva from the cotton pad. Multiplex LSG sensors were prepared by immobilizing the BZD antibody on WE1s, the COC antibody on WE2s, and the AMP antibody on WE3s for 16 h.

Three different analyte concentrations for COC (100, 500 pg/mL, and 1.0 μg/mL), AMP (500 pg/mL, 25 ng/mL, and 1.0 μg/mL), and BZD (100, 500 pg/mL, and 500 ng/mL) were selected to evaluate the trend of the oxidation signal with increasing concentrations. Another set of multiplex sensors was prepared to test the effect of pregabalin (Lyrica), an anticonvulsant medication used to treat epilepsy and neuropathic pain. The same analyte concentrations were mixed with Lyrica in a 4:1 ratio to simulate real use cases and to observe the effect of Lyrica in the measurement media. For the clinical study, multi-LSG sensors were prepared by immobilizing the BZD antibody on WE1s, the COC antibody on WE2s, and the AMP antibody on WE3s. We received clinical saliva samples from Tinaztepe Buca Hospital (Izmir, Turkey). Healthy saliva, AMP-spiked healthy saliva, MET patient saliva, and AMP-spiked MET patient saliva samples were incubated on each WE. The interference effect of MET with and without the presence of AMP was observed, and the accuracy of multi-LSG sensor was tested with real samples.

## Results and Discussion

### Surface Modification and Characterization of the Multiplex Sensor

**Figure 1:**
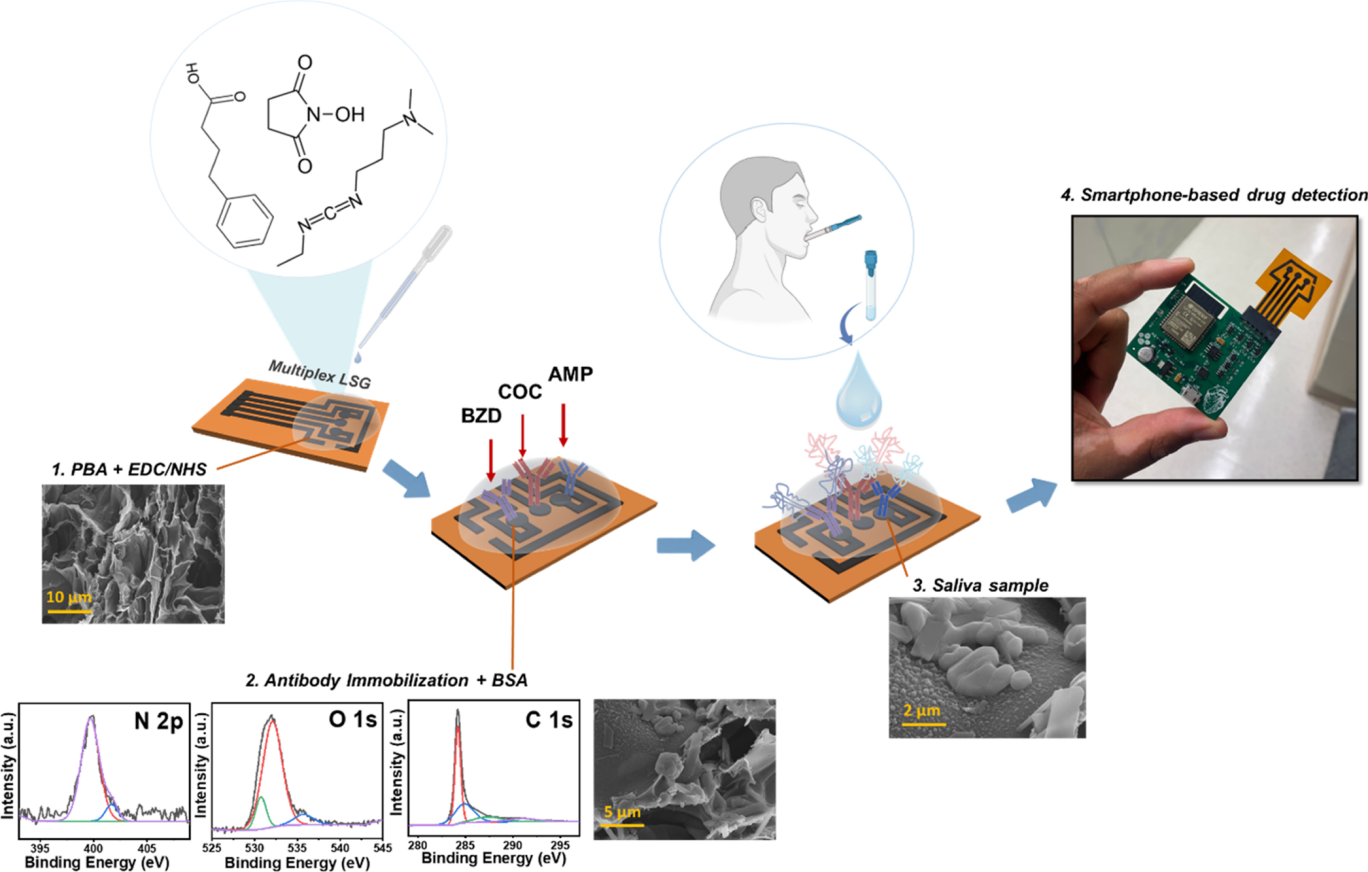
Schematic representation of the multiplex LSG sensor’s fabrication steps, including scanning electron microscopy (SEM) images at a scale bar of 2, 5, and 10 μm and X-ray photoelectron spectroscopy (XPS) data containing C 1s, O 1s, and N 1s spectra of the sensor.

Figure 1 summarizes the surface modification steps of the multiplex sensor. As the first step, PBA was incubated on the graphene surface of the working electrode for 1 h to provide the necessary active groups for further functionalization. The aromatic groups of PBA form π–π stacking through existing aromatic groups of the graphene surface. EDC/NHS binders were dropped and cast onto a PBA-modified surface. First, the carboxylic group from pyrenebutyric acid is activated by EDC, by forming an intermediate O-acylisourea compound. In the presence of NHS, this intermediate forms an NHS ester. Then, antibodies were immobilized onto the electrode surface by their amine ends binding the EDC/NHS-modified surface. The ester intermediate reacts spontaneously with primary amines from antibodies forming amides.^48^ The access groups were cleaned from the surface, and the electrode surface was blocked with BSA for 1 h to avoid nonspecific bonds. Finally, COC, AMP, and BZD analytes were incubated separately on the developed sensors for 30 min. XPS and SEM characterization analyses were performed to observe the LSG surface for each immobilization step. After each immobilization step, the working electrode surface remained rough and flakey, as shown in Figures S2 and S3. The addition of bulky structures onto the LSG surface after antibody immobilization was visually observed. The elemental characterization was performed after each immobilization step to show the chemical changes happening on the surface. Figures S6 and S7 demonstrate the successful incorportion of antibodies as shown by the increase in carbon and nitrogen content due to the presence of H_3_C–N and O–CH_3_ C=O groups of COC and BZD and of C–NH_2_ groups of AMP, as shown in Figure S4. Aromatic groups of each antibody also contribute to the increase in carbon content.

### Single-Antibody Sensor Performance of AMP, COC, and BZD Sensors

**Figure 2:**
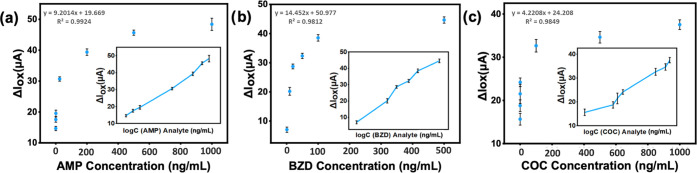
(a) Sensitivity valuation of PBS/LSG sensors. Oxidation response change in DPV signals of (a) AMP, (b) BZD and (c) COC antibodies immobilized on working electrode surface for different concentrations of analytes. Three scans used for error bar calculation are collected from three different immunosensors. Measurements were performed using the PALMSENS potentiostat in 5.0 mM [Fe(CN)_6_]^3–/4–^ containing 0.1 M PBS and 0.1 M KCl. The inset shows the relationship between the ΔIox and the logarithm of the concentration of the (a) AMP, (b) BZD and (c) COC analytes.

Before testing the performance of multiplex sensors, the performance of single-antibody sensors (singlet LSG sensors) having one reference, one working, and one counter electrode was evaluated. The fabrication procedure and singlet and multiplex LSG sensors are represented in Figure S8. Antibodies of COC, AMP, and BDZ were incubated on working electrodes to investigate antibody–analyte interaction individually. Different concentrations of COC, AMP, and BZD analytes were kept on singlet antibody-incubated LSG sensors for 1 h. Then, differential pulse voltammograms were recorded to evaluate the performance of each sensor. The oxidation current difference between before and after analyte incubation in the DPV signal was taken into account to evaluate the sensor performance regarding analyte–antibody interactions. For singlet LSG sensor evaluation, electrochemical measurements were performed by using a commercial PalmSens potentiostat. First, the antibody concentration used in sensor preparation was optimized for each illicit drug individually. 25 μg/mL AMP antibody, 25 μg/mL BZD antibody, and 200 ng/mL COC antibody were selected as optimal antibody concentrations considering the best sensor performance. Then, the incubation solution volume was tested between 4 and 10 μL to be able to modify each working electrode individually,

The incubation time of the analyte was tested between 30 min and 2 h, and 30 min was found to be the optimal incubation time for analytes. After the optimizations, the analyte detection range for each sensor was investigated by using the sensors individually prepared with optimized antibody amounts and incubation conditions. The sensitivity of each antibody drug was observed in different ranges depending on the antibody. Each singlet LSG sensor was responsive in the range of 1.0 pg/mL–500 ng/mL for BDZ, 1.0 pg/mL–1.0 μg/mL for COC, and 250 pg/mL–1.0 μg/mL for AMP. The logarithmic relation between the decrease in oxidation response and the analyte concentration incubated on each sensor is shown in Figure 2. The LODs of AMP, BZD, and COC have been calculated as 9.7, 9.0, and 4.3 ng/mL, respectively, (LOD = 3.3 σ/S). Thus, the compatibility of each antibody–analyte was proved. Following this validation, the rest of the sensor performance experiments with standards and real samples was carried out using the KAUSTat potentiostat.

### Simultaneous Detection of Different Concentrations of Drugs

As the first performance evaluation, three working areas of the multiplex sensor, namely, WE1, WE2, and WE3, were immobilized with antibodies of the same drug. Corresponding analytes were tested at different concentrations to be able to detect oxidation current differences with repeated DPV measurements performed by the KAUSTat potentiostat. The app control including connection screen, control screen, and data visualization screen is given in Figures S9 and S10. 1.0, 100, and 500 ng/mL AMP analytes were tested on the AMP multiplex sensor; 1.0, 100, and 500 ng/mL COC analytes were tested on the COC multiplex sensor; and 1.0 pg/mL, 200, and 500 ng/mL BZD analytes were tested on the benzodiazepine multiplex sensor. Oxidation current differences (ΔIox) increased with the increase of each drug concentration. In total, three sensors were prepared for each drug test. First, WE1 was tested for 1.0 ng/mL AMP while keeping WE2 at a constant 10 ng/mL AMP and WE3 empty as blank. Two more AMP sensors were prepared by immobilizing 100 and 500 ng/mL onto WE1 while keeping WE2 at a constant 10 ng/mL AMP and WE3 empty as blank.

**Figure 3:**
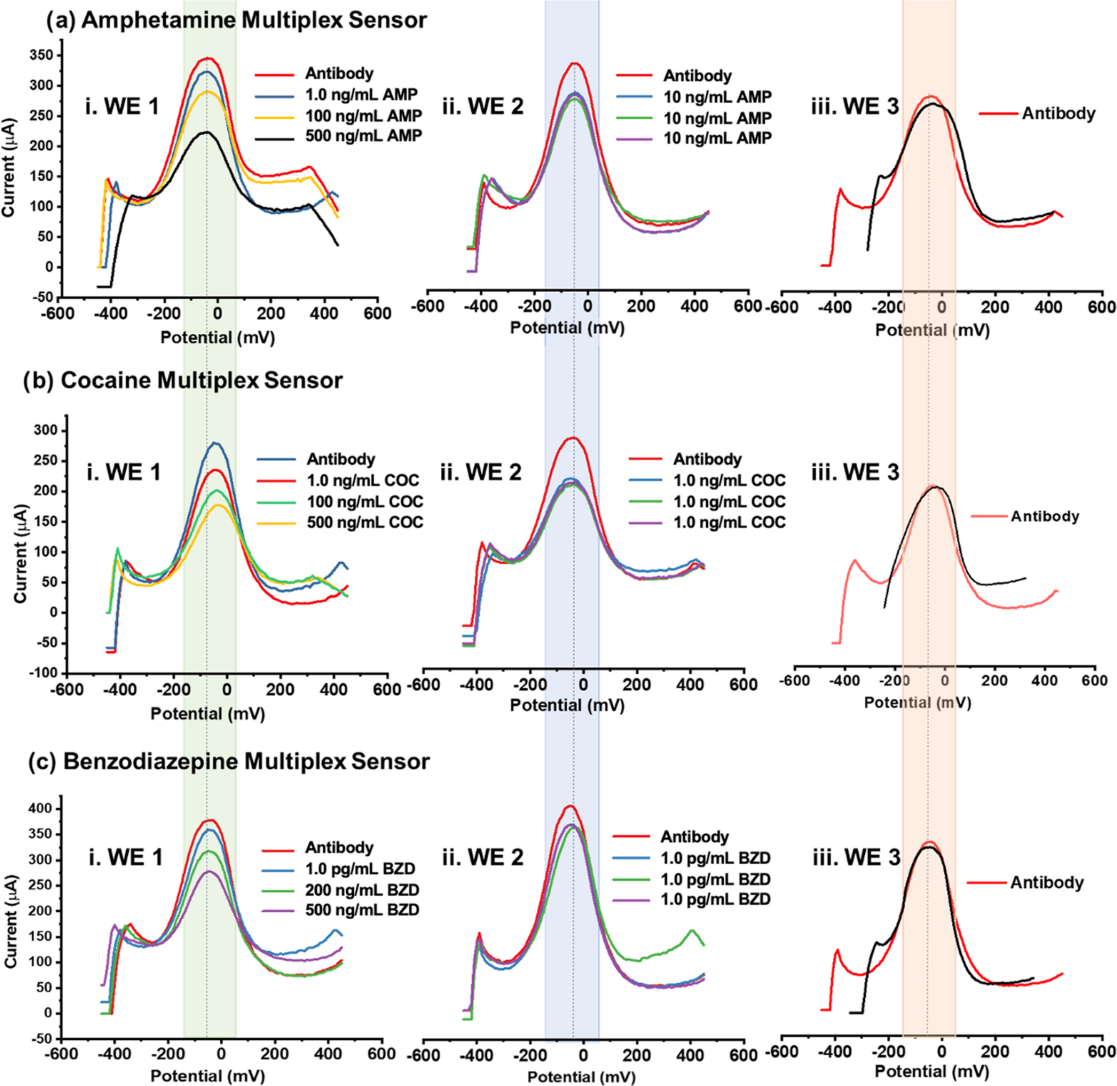
Performance test of each drug on the multiplex platform. Simultaneous DPV responses of (a) multiplex sensors having AMP antibody immobilized for i. 1.0, 100, and 500 ng/mL AMP on WE1; ii. 10 ng/mL on WE2; and iii. the blank sensor as WE3; (b) multiplex sensors having COC antibody immobilized for i. 1.0, 100, and 500 ng/mL COC on WE1; ii. 1.0 ng/mL on WE2; and iii. the blank sensor as WE3; and (c) multiplex sensors having benzodiazepine antibody immobilized for i. 1.0 pg/mL, 200, and 500 ng/mL BZD on WE1; ii. 1.0 pg/mL on WE2; and iii. the blank antibody sensor (red) and after 0.1 M PBS incubation (black) on WE3. Measurements were performed using the KAUSTat potentiostat in 5.0 mM [Fe(CN)_6_]^3–/4–^ containing 0.1 M PBS and 0.1 M KCl.

By doing so, the distinguished current response of each analyte concentration was observed despite having the same redox probe media during simultaneous measurements for a single antibody–analyte couple. The same test was repeated with three multiplex sensors for 1.0, 100, and 500 ng/mL COC analyte on WE1 surfaces while keeping WE2 at a constant 1.0 ng/mL COC and WE3 blank. The third working electrode was kept as a blank reference by incubating PBS solution during analyte incubations on the rest of working electrodes. Finally, the oxidation current differences of three multiplex BZD sensors were recorded while keeping WE1s as 1.0 pg/mL, 200, and 500 ng/mL BZD while WE2 at a constant 1.0 pg/mL BZD and WE3 blank. Overall, the oxidation response was observed to be following a decreasing trend after the drug analyte incubations with increasing concentrations, without having a significant error to disturb the signal (Figure 3)

### Simultaneous Detection of Different Illicit Drugs in Complex Media

**Figure 4:**
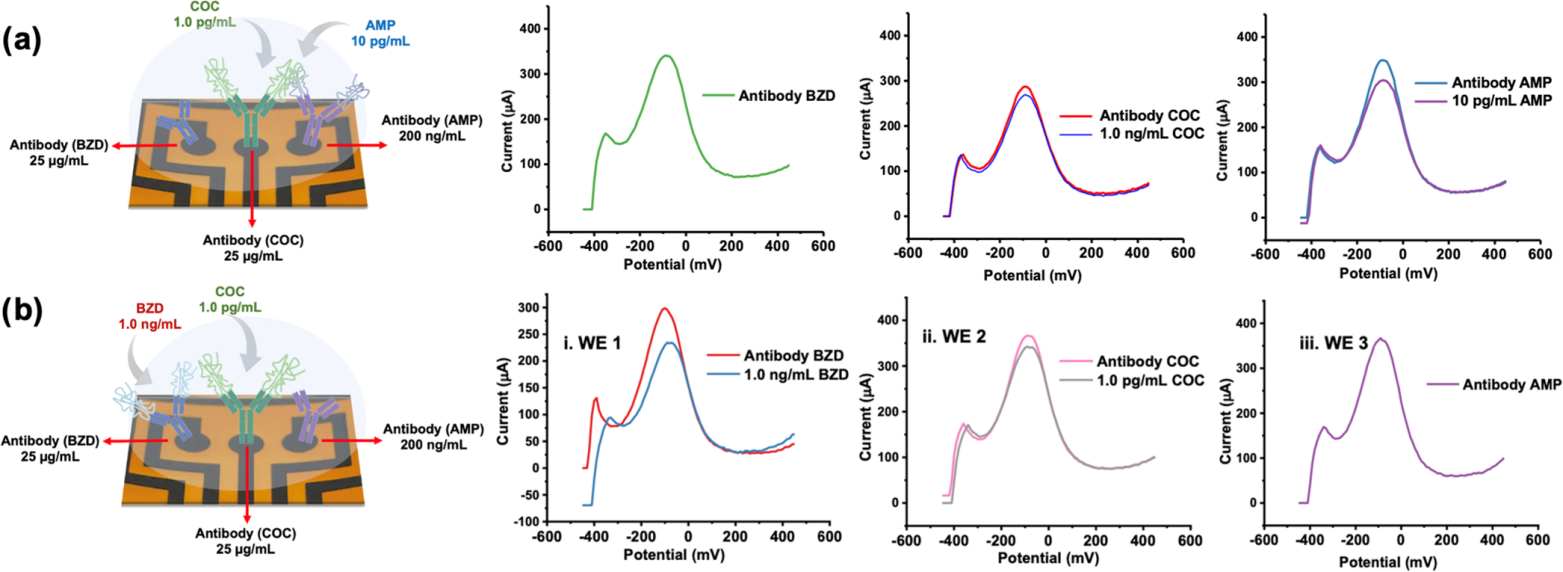
Performance test of multiplex sensors in complex media. The BZD antibody was immobilized on WE1, the COC antibody on WE2, and the AMP antibody on WE3 for each sensor. (A) Simultaneous DPV responses of a multiplex sensor for blank WE1 (RSD: 1.2%), 1.0 pg/mL COC on WE2 (RSD: 2.6%), and 10 pg/mL AMP on WE3 (RSD: 1.8%). (B) DPV responses of a multiplex sensor for 1.0 ng/mL BZD WE1 (RSD: 3.1%), 1.0 pg/mL COC on WE2 (RSD: 3.9%), and blank WE3 (RSD: 1.5%). Measurements were performed using the KAUSTat potentiostat in 5.0 mM [Fe(CN)_6_]^3–/4–^ containing 0.1 M PBS and 0.1 M KCl.

Following the tests of multiplex sensors prepared with the same antibody, the effect of immobilizing different antibodies onto each WE was tested as the next step. The multiplex sensor performance having different antibodies on each WE was evaluated by following two scenarios: (i) the sensor was prepared by keeping WE1 as blank with PBS incubation while immobilizing 1.0 pg/mL COC and 10 pg/mL AMP on WE2 and WE3 areas, respectively, as illustrated in Figure 4a.

All antibody and analyte incubations were performed with small amounts and directly onto the specific working areas without interfering with each other. (ii) The sensor was prepared by immobilizing 1.0 ng/mL BZD and 1.0 pg/mL COC on WE1 and WE2, respectively, while keeping PBS incubated on WE3 as blank, without any analyte (Figure 4b). Similarly, immobilization steps were performed isolated from each other. The sensor performances were recorded in 5.0 mM [Fe(CN)_6_]^3–/4–^ containing 0.1 M PBS and 0.1 M KCl by the KAUSTat potentiostat measuring all three working areas simultaneously through the smartphone application. The results show that multiple antibodies were successfully immobilized resulting in specific bindings of each drug analyte in the same sensing platform.

### Simultaneous Detection and Selectivity in Real Saliva

**Figure 5:**
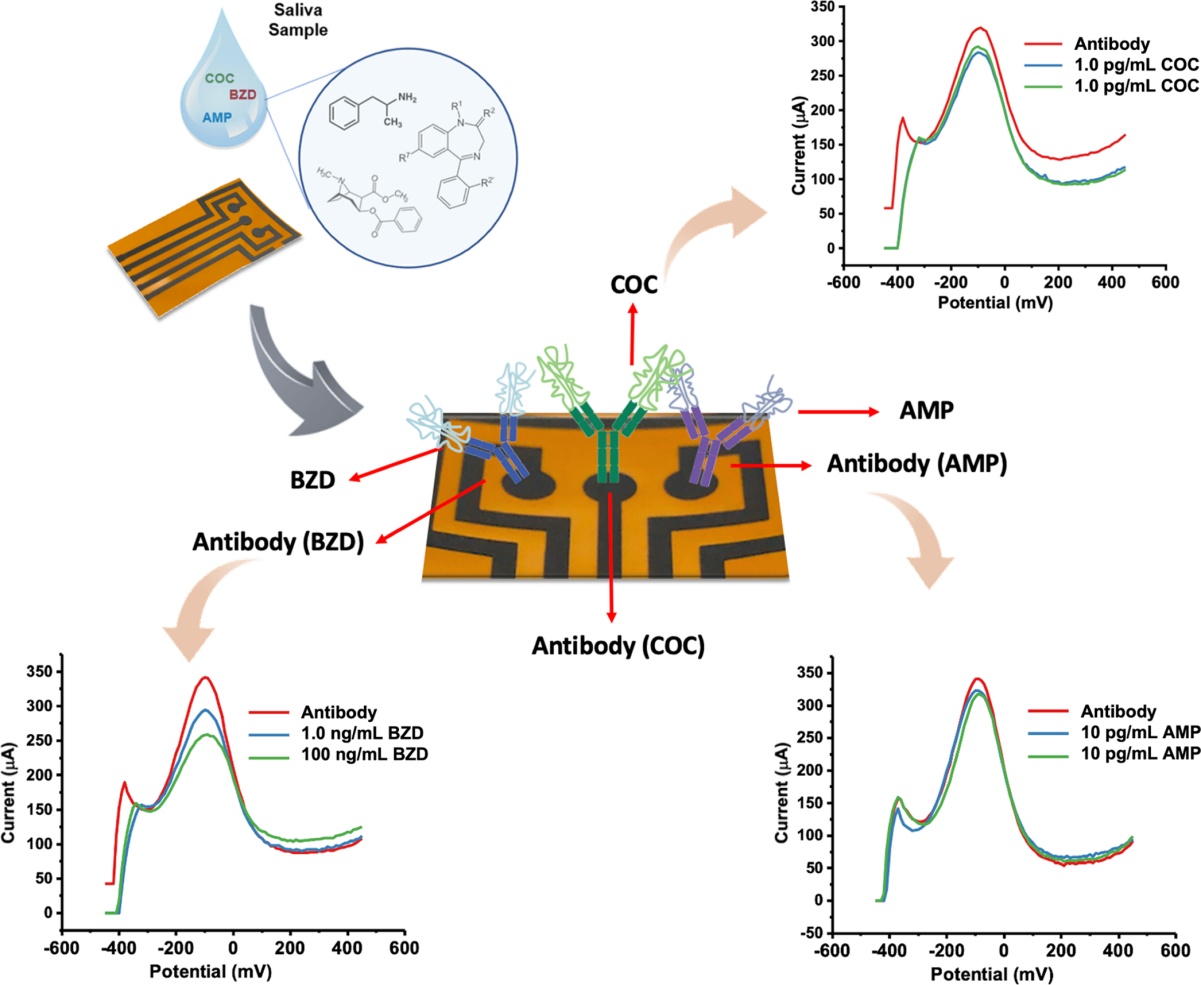
Simultaneous detection of three illicit drugs on a single platform. The DPV responses of two multiplex sensors having BZD antibody immobilized on WE1, COC antibody immobilized on WE2, and AMP antibody immobilized on WE3. The response of the first multiplex sensor was recorded after introducing a spiked saliva solution having a 1.0 pg/mL COC, 1.0 ng/mL BDZ, and 10 pg/mL AMP mixture. The second multiplex sensor was exposed to a spiked saliva solution having a 1.0 pg/mL COC, 100 ng/mL BDZ, and 10 pg/mL AMP mixture. Measurements were performed using the KAUSTat potentiostat in 5.0 mM [Fe(CN)6]^3–/4–^ containing 0.1 M PBS and 0.1 M KCl.

The performance of the multiplex sensors was tested in spiked real saliva samples to simulate the real complex media. Different combinations of drug analytes were tested to observe the selectivity of each antibody in complex media. BZD antibody was immobilized on WE1s, the COC antibody immobilized on WE2s, and the AMP antibody immobilized on WE3s for each sensor. In the first case, a spiked saliva solution having a mixture of 1.0 pg/mL COC, 1.0 ng/mL BZD, and 10 pg/mL AMP was incubated on the surface to observe simultaneous detection of targeted drugs in the presence of all drugs in different concentrations. Then, the concentration of the BZD analyte was changed to 100 ng/mL, while other drug concentrations remained the same. The mixture was tested, and the oxidation current of the BZD sensor dropped, with a greater concentration of BZD, while AMP and COC sensors displayed the same current difference. Figure 5 shows the surface modification of the multiplex sensor and oxidation current differences for both cases. Both sensors successfully detected target analytes and demonstrate that WE1 detects BZD, WE2 detects COC, and WE3 detects AMP in spiked saliva mixtures and yield the same current change for selected concentration without any significant cross-reactivity. The reliability of the multisensing platform was tested with healthy and MET patient saliva samples. The samples were incubated on WEs directly and after being spiked with AMP. As expected, healthy saliva showed no significant change in current responses related to absence of drugs. AMP-spiked saliva samples showed higher current differences on the AMP-specific WE, while the current difference of BZD and COC WEs remained the same. The AMP-spiked MET saliva sample resulted in the highest oxidation current difference, approx. 3 times greater than the MET saliva sample and 1.5 times greater than the AMP-spiked healthy saliva sample, as shown in Figure 7. The reason for this cross-reactivity can be explained by the similarity of AMP and MET structures. Other reasons for the high current change of MET saliva might be the impurity of street samples and multiple types of drug consumption in the same period. Overall, the identification of AMP was still possible with the proposed diagnostic platform, even in the presence of MET as an interference in real sample media.

**Figure 6:**
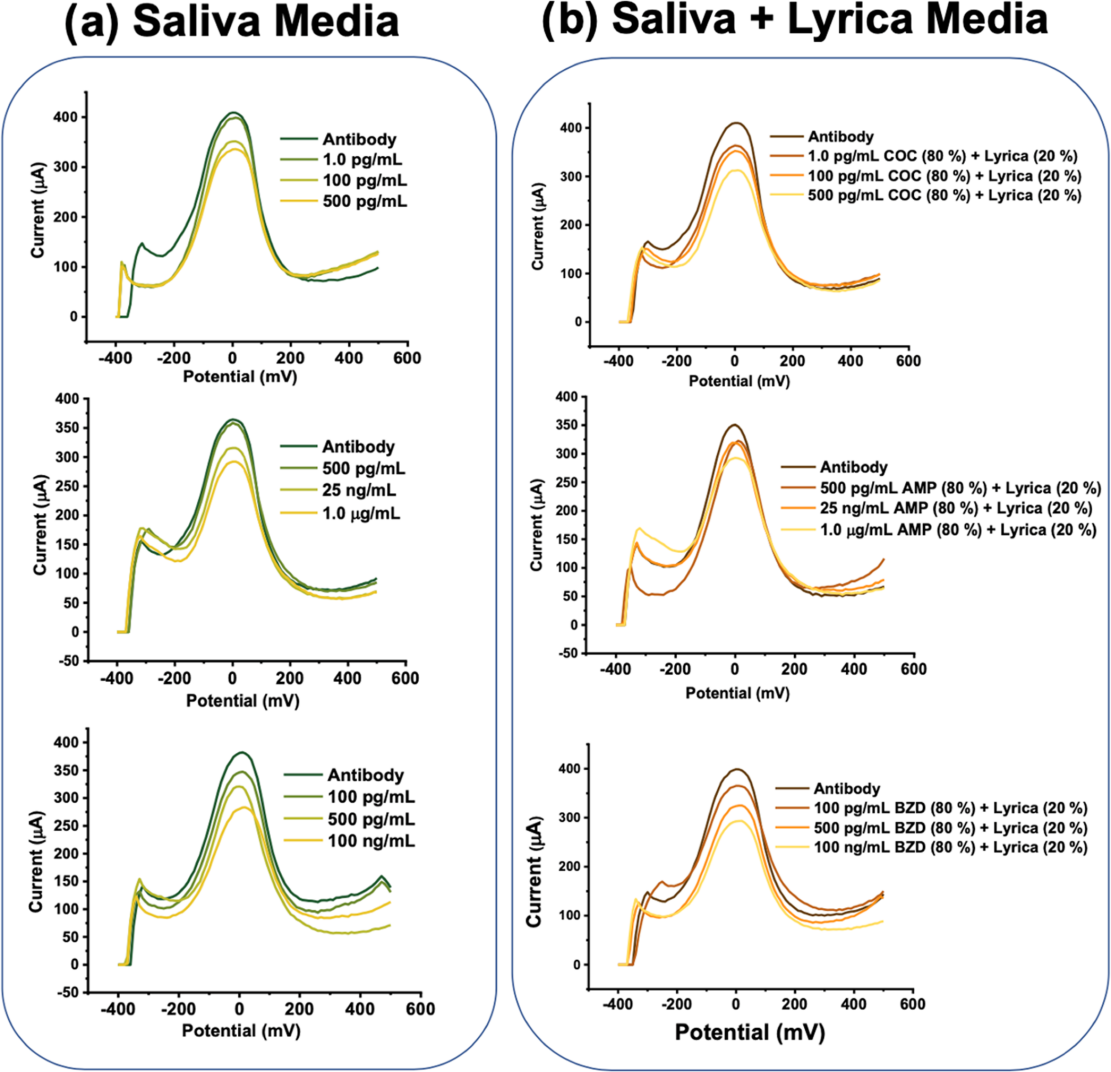
Performance test of multiplex sensors in real saliva. Simultaneous DPV responses recorded by the KAUSTat potentiostat after (a) COC (1.0, 100, 500 pg/mL) immobilization on first WEs, AMP (500 pg/mL, 25 ng/mL, 1.0 μg/mL) immobilization on second WEs, and BZD (100, 500 pg/mL, 100 ng/mL) immobilization on third WEs. (b) COC (80%) (1.0, 100, 500 pg/mL) + Lyrica (20%) immobilization on first WEs, AMP (80%) (500 pg/mL, 25 ng/mL, 1.0 μg/mL) + Lyrica (20%) immobilization on second WEs, and BZD (80%) (100, 500 pg/mL, 100 ng/mL) + Lyrica (20%) immobilization on third WEs. All solutions were prepared in real saliva. Measurements were performed using the KAUSTat potentiostat in 5.0 mM [Fe(CN)_6_]^3–/4–^ containing 0.1 M PBS and 0.1 M KCl.

**Figure 7:**
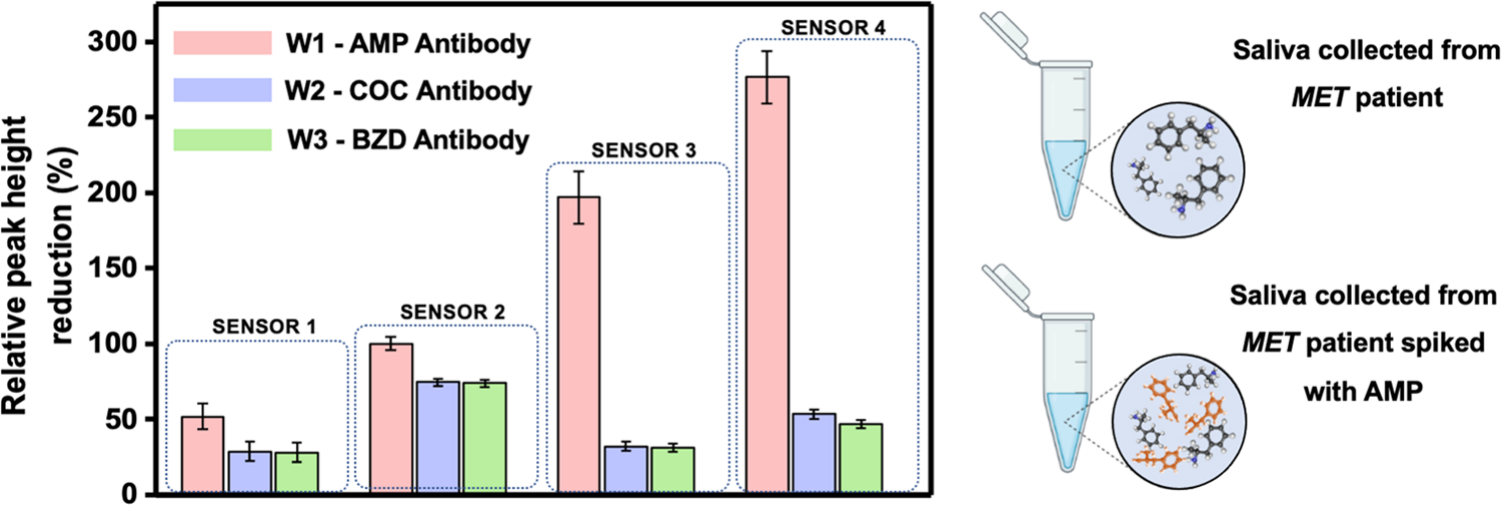
Relative peak height reduction calculated from the DPV signals of multiplex sensors having COC, AMP, and BZD antibodies. The saliva sample was collected from a real patient admitted to the clinic for methamphetamine (MET) use. Sensors 1, 2, 3, and 4 represent the detection of blank healthy saliva, blank MET patient saliva, AMP-spiked saliva, and AMP-spiked MET patient saliva, respectively. Measurements were performed using the KAUSTat potentiostat in 5.0 mM [Fe(CN)6]^3–/4–^ containing 0.1 M PBS and 0.1 M KCl.

### Effect of Mixing Analgesic Medications with Illicit Drugs

**Figure 8:**
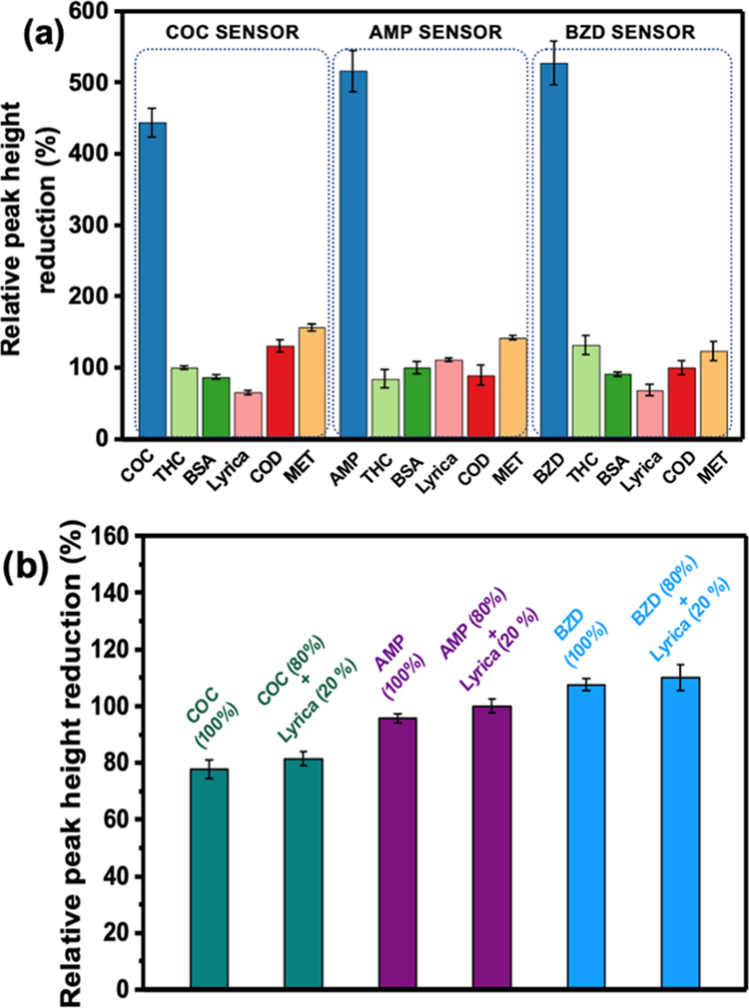
Selectivity study of the multiplex sensors. (a) Relative peak height reduction calculated from the DPV signals of multiplex sensors having COC, AMP, and BZD antibodies immobilized for tetrahydrocannabinol (THC), bovine serum albumin (BSA), pregabalin (Lyrica), methamphetamine (MET), and codeine (COD) as interferences (100 ng/mL each). (b) Relative peak height reduction calculated from the DPV signals of multiplex sensors having COC, AMP, and BZD antibodies immobilized for COC (100%), COC (80%) + Lyrica (20%), AMP (100%), AMP (80%) + Lyrica (20%), BZD (100%), and BZD (80%) + Lyrica (20%). Three scans used for error bar calculation are collected from three different immunosensors.

The use of illicit drugs in combination with analgesics has increased in recent years. After validation, the sensitivity of our complex detection system and the effect of having Lyrica in the detection media were evaluated to further test the reliability of the POC system. For all multiplex sensors, COC, AMP, and BZD were immobilized onto WE1, WE2, and WE3, respectively. AMP-spiked saliva samples were incubated on the AMP multiplex sensor, COC-spiked saliva samples incubated on the COC multiplex sensor, and BZD-spiked saliva samples incubated on the benzodiazepine multiplex sensor. Figure 6a shows results obtained from the three multiplex sensors detecting different concentrations of COC, AMP, and BZD on different working electrodes in the same real saliva media. The same set of sensors was prepared to replace 20% of each media with Lyrica. The same analyte concentration of each drug was mixed with Lyrica at a 4:1 ratio. The trend between drug analyte concentration and oxidation current responses recorded in the DPV measurements shown in Figure 6 proves that the developed detection platform provides accurate results in real saliva in the presence of analgesic medication. Figure 8b shows the oxidation current decreases of the sensors after introducing saliva samples to the surface. Each drug analyte, with and without the addition of Lyrica, was incubated in the working area. Results are compared side by side in Figure 8b, which clearly shows that the samples with Lyrica resulted in similar current differences. Though there is an increase of 10–20% observed in samples with Lyrica, the cross-reactivity can be considered negligible in terms of targeted drug detection.

### Selectivity of the Multiplex Sensor

Potential interferants such as methamphetamine, THC, Lyrica codeine, and BSA were tested to evaluate the sensor reliability. The concentrations were fixed at 100 nM for each drug in 0.1 M PBS. First, multiplex LSG sensors were functionalized with PBA/EDC/NHS. Then, all three working electrodes of a multiplex sensor were modified with COC, AMP, or BZD antibodies. After interferants and target analytes were incubated individually on modified sensors, oxidation current responses were recorded by the KAUSTat potentiostat simultaneously. Figure 8a shows DPV signals corresponding to the oxidation current change of interferants incubated on multiplex sensors. The target drug analytes show an approximately 3-fold, 4-fold, and 4.5-fold increase in current responses compared to interferences, as expected. This trend proves that our multiplex sensing platform is highly selective for AMP, BZD, and COC. For all of the antibody surfaces, methamphetamine shows slight cross-reactivity compared to other interferences. This behavior is most likely related to the structural similarity to AMP.

Table S1 summarizes the existing biosensors for the three analytes investigated in this study: COC, AMP, and BZD. There is a wide range of materials, including gold nanoparticles, carbon nanotubes, graphene, and polymers, among others, that have been used for sensor fabrication. Working with complex biological media such as saliva, sweat, or blood requires specific attention in terms of the elimination of cross-reactivity. A limited amount of sensing platforms exists for the detection of multiple drugs. A multiplex biosensor has been developed for the simultaneous detection of THC, benzoylecgonine, and morphine.^48^ A dual sensor has been reported for the simultaneous detection of amphetamine and methamphetamine.^49^ Several sensing platforms have been reported for the nonsimultaneous detection of benzodiazepine variations (e.g., clonazepam, diazepam, or oxazepam). However, these studies have used single working electrode with one reference and counter electrode. In the literature, there are biosensors with a single working area, but there is no study reporting the detection of these three illicit drugs simultaneously. We have successfully developed a POC platform with negligible cross-reactivity for multiple drug detection. Though lateral flow assays are currently advantageous on-site drug detection platforms, they might suffer from low sensitivity, leading to false positive-negative results. Moreover, LFAs provide qualitative and semiquantitative results, rather than providing quantitative values regarding drug quantity in the system. The proposed sensor serves a role of to be the first step toward a noninvasive quantitative platform for drug diagnostics.^50^ For the first time, in this study, the custom-made potentiostat, KAUSTat, has proved its potential to be a POC screening tool for simultaneous analyte detection in real saliva media. We acknowledge that further improvements are necessary to enhance the sensitivity of the device to perform better quantification; however, considering the increase in drug abuse rates during the recent COVID-19 pandemic, the proposed multiplex LSG sensor with KAUSTat integration has the potential to increase the screening rate and control the abuse through both personal and clinical use.^51^

## Conclusions

A smartphone-based multiplex LSG sensor for the simultaneous detection of AMP, COC, and BZD in real saliva samples was successfully developed. Multiplex electrodes were individually modified to detect illicit drugs with LODs of AMP, BZD, and COC calculated as 4.3, 9.7, and 9.0 ng/mL, respectively. First, the compatibility between antibodies and drug analytes was tested individually by using single LSG electrodes. Later, the voltammetric behavior of having different antibodies in the same measurement media was tested. Both synthetic and real saliva samples were used to test and validate the performance of the multiplex sensors. Healthy and MET patient saliva samples were used for the clinical study. The real samples were spiked with the desired amounts of drug, and as a crucial requirement, complex saliva media having different concentrations of different drugs were successfully tested. The multiplex platform proved its potential for both the drug identification in complex saliva media and the successful quantification of each drug. Having a high selectivity, the multiplex LSG platform has a high potential to be implemented in real screening fields. The easy-to-use POC platform was created with multiplex sensor integration into a handmade potentiostat with a wireless connection. This multiplex sensing platform holds the potential of being used for rapid and on-site illicit-drug detection as well as various clinical applications in the near future.

## Supporting Information Available

Laser scribing; fabrication process, electrochemical measurements; statistical analysis; characterizations; chemical structures of AMP, BZD, and COC; KAUSTat; app visualization, analytical features; and summary of existing biosensing systems for AMP, BZD, and COC (PDF)

## References

[ref1] Ren S.; Zeng J.; Zheng Z.; Shi H. Perspective And Application Of Modified Electrode Material Technology In Electrochemical Voltammetric Sensors For Analysis And Detection Of Illicit Drugs. Sens. Actuators A 2021, 329, 112821 10.1016/J.Sna.2021.112821.

[ref2] Nawi A. M.; Ismail R.; Ibrahim F.; Hassan M. R.; Manaf M. R. A.; Amit N.; Ibrahim N.; Shafurdin N. S. Risk And Protective Factors Of Drug Abuse Among Adolescents: A Systematic Review BMC Public Health, 21 2088 10.1186/S12889-021-11906-2. PMC8590764 34774013

[ref3] The United Nations Office on Drugs and Crime (UNODC), World Drug Report 2021, 2021.

[ref4] Adepu S.; Ramakrishna S. Controlled Drug Delivery Systems: Current Status And Future Directions. Molecules 2021, 26, 5905 10.3390/Molecules26195905. 34641447 PMC8512302

[ref5] De Rycke E.; Stove C.; Dubruel P.; De Saeger S.; Beloglazova N. Recent Developments In Electrochemical Detection Of Illicit Drugs In Diverse Matrices. Biosens. Bioelectron. 2020, 169, 112579 10.1016/J.Bios.2020.112579. 32947080

[ref6] Scanferla D. T. P.; Lini R. S.; Marchioni C.; Mossini S. A. G. Drugs Of Abuse: A Narrative Review Of Recent Trends In Biological Sample Preparation And Chromatographic Techniques. Forensic Chem. 2022, 30, 100442 10.1016/J.Forc.2022.100442.

[ref7] Wagieh N. E.; Abbas S. S.; Abdelkawy M.; Abdelrahman M. M. Spectrophotometric And Spectrodensitometric Determination Of Triamterene And Xipamide In Pure Form And In Pharmaceutical Formulation. Drug Test. Anal. 2010, 2, 113–121. 10.1002/dta.92. 20878892

[ref8] Hill S. L.; Thomas S. H. L. Drugs Of Abuse. Medicine 2016, 44, 160–169. 10.1016/J.Mpmed.2015.12.030.

[ref9] Ali I.; Suhail M.; Alothman Z. A.; Abdulrahman A.; Aboul-Enein H. Y. Drug Analyses In Human Plasma By Chromatography. In Handbook Of Analytical Separations; Hempel G., Ed.; Elsevier Science B.V, 2020; Vol. 7, Chapter 2, pp 15–46.

[ref10] Childress A. C. Stimulants. Child Adolesc. Psychiatr. Clin. North Am. 2022, 31, 373–392. 10.1016/J.Chc.2022.03.001. 35697391

[ref11] Couch G. A.; White M. P.; de Gray L. E. Central Nervous System Stimulants: Basic Pharmacology And Relevance To Anaesthesia And Critical Care. Anaesth. Intensive Care Med. 2020, 21, 503–511. 10.1016/J.Mpaic.2020.07.005.

[ref12] Maisto S. A.; Galizio M.; Connors G. J. Drug Use And Misuse; Holt, Rinehart And Winston, 1991.

[ref13] Schmitz A. Benzodiazepine Use, Misuse, And Abuse: A Review. Mental Health Clin. 2016, 6, 120–126. 10.9740/Mhc.2016.05.120. PMC6007645 29955458

[ref14] Loftsson T. 1,4-Benzodiazepines: Chemical Stability And Cyclodextrin Solubilization. J. Drug Delivery Sci. Technol. 2021, 66, 102936 10.1016/J.Jddst.2021.102936.

[ref15] Li M.; Wessinger W. D.; Mcmillan D. C. Effects Of Amphetamine-Cns Depressant Combinations And Of Other Cns Stimulants In Four-Choice Drug Discriminations. J. Exp. Anal. Behav. 2005, 84, 77–97. 10.1901/Jeab.2005.09-04. 16156138 PMC1243898

[ref16] Dagar M.; Yadav S.; Sai V. V. R.; Satija J.; Bhatia H. Emerging Trends In Point-Of-Care Sensors For Illicit Drugs Analysis. Talanta 2022, 238, 123048 10.1016/J.Talanta.2021.123048. 34801905

[ref17] Ahmed S. R.; Chand R.; Kumar S.; Mittal N.; Srinivasan S.; Rajabzadeh A. R. Recent Biosensing Advances In The Rapid Detection Of Illicit Drugs. TrAC, Trends Anal. Chem. 2020, 131, 116006 10.1016/J.Trac.2020.116006.

[ref18] Sanli S.; Moulahoum H.; Ghorbanizamani F.; Celik E. G.; Timur S. Ultrasensitive Covalently-Linked Aptasensor For Cocaine Detection Based On Electrolytes-Induced Repulsion/Attraction Of Colloids. Biomed. Microdevices 2020, 22, 51 10.1007/S10544-020-00507-2. 32748213

[ref19] Hadler N. L.; Hairston I. S.; Conroy D. A. Insomnia Due To Drug Or Substance Abuse And Dependence. In Reference Module In Neuroscience And Biobehavioral Psychology; Elsevier, 2021.

[ref20] Watson R. Cocaine Use Rises In Europe While Overall Drug Use Levels Out. BMJ 2007, 335, 1117 10.1136/Bmj.39412.365718.Db. PMC2099547 18048528

[ref21] Greene S. L.; Kerr F.; Braitberg G. Review Article: Amphetamines And Related Drugs Of Abuse. Emerg. Med. Australas. 2008, 20, 391–402. 10.1111/J.1742-6723.2008.01114.X. 18973636

[ref22] Schmitz A. Benzodiazepine Use, Misuse, And Abuse: A Review. Mental Health Clin. 2016, 6, 120–126. 10.9740/mhc.2016.05.120. PMC6007645 29955458

[ref23] Guler E.; Sengel T. Y.; Gumus Z. P.; Arslan M.; Coskunol H.; Timur S.; Yagci Y. Mobile Phone Sensing Of Cocaine In A Lateral Flow Assay Combined With A Biomimetic Material. Anal. Chem. 2017, 89, 9629–9632. 10.1021/Acs.Analchem.7b03017. 28831804

[ref24] Tamama K. Advances In Drugs Of Abuse Testing. Clin. Chim. Acta 2021, 514, 40–47. 10.1016/J.Cca.2020.12.010. 33333045

[ref25] Mani V.; Beduk T.; Khushaim W.; Ceylan A. E.; Timur S.; Wolfbeis O. S.; Salama K. N. Electrochemical Sensors Targeting Salivary Biomarkers: A Comprehensive Review. TrAC, Trends Anal. Chem. 2021, 135, 116164 10.1016/J.Trac.2020.116164.

[ref26] Parrilla M.; Joosten F.; De Wael K. Enhanced Electrochemical Detection Of Illicit Drugs In Oral Fluid By The Use Of Surfactant-Mediated Solution. Sens. Actuators, B 2021, 348, 130659 10.1016/J.Snb.2021.130659.

[ref27] Guinan T.; Ronci M.; Kobus H.; Voelcker N. H. Rapid Detection Of Illicit Drugs In Neat Saliva Using Desorption/Ionization On Porous Silicon. Talanta 2012, 99, 791–798. 10.1016/J.Talanta.2012.07.029. 22967625

[ref28] Trefz P.; Kamysek S.; Fuchs P.; Sukul P.; Schubert J. K.; Miekisch W. Drug Detection In Breath: Non-Invasive Assessment Of Illicit Or Pharmaceutical Drugs. J. Breath Res. 2017, 11, 024001 10.1088/1752-7163/Aa61bf. 28220762

[ref29] Denia A.; Esteve-Turrillas F. A.; Armenta S. Analysis Of Drugs Including Illicit And New Psychoactive Substances In Oral Fluids By Gas Chromatography-Drift Tube Ion Mobility Spectrometry. Talanta 2022, 238, 122966 10.1016/J.Talanta.2021.122966. 34857341

[ref30] Anzar N.; Suleman S.; Parvez S.; Narang J. A Review On Illicit Drugs And Biosensing Advances For Its Rapid Detection. Process Biochem. 2022, 113, 113–124. 10.1016/J.Procbio.2021.12.021.

[ref31] Metternich S.; Zörntlein S.; Schönberger T.; Huhn C. Ion Mobility Spectrometry As A Fast Screening Tool For Synthetic Cannabinoids To Uncover Drug Trafficking In Jail Via Herbal Mixtures, Paper, Food, And Cosmetics. Drug Test. Anal. 2019, 11, 833–846. 10.1002/dta.2565. 30610761

[ref32] Liu C. M.; He H. Y.; Xu L.; Hua Z. D. New Qualitative Analysis Strategy For Illicit Drugs Using Raman Spectroscopy And Characteristic Peaks Method. Drug Test. Anal. 2021, 13, 720–728. 10.1002/dta.2963. 33142047

[ref33] Kranenburg R. F.; Ou F.; Sevo P.; Petruzzella M.; de Ridder R.; van Klinken A.; Hakkel K. D.; van Elst D. M. J.; van Veldhoven R.; Pagliano F.; et al. On-Site Illicit-Drug Detection With An Integrated Near-Infrared Spectral Sensor: A Proof Of Concept. Talanta 2022, 245, 123441 10.1016/J.Talanta.2022.123441. 35405444

[ref34] Zhong Y.; Huang K.; Luo Q.; Yao S.; Liu X.; Yang N.; Lin C.; Luo X. The Application Of A Desktop Nmr Spectrometer In Drug Analysis. Int. J. Anal. Chem. 2018, 2018, 3104569 10.1155/2018/3104569. 30327671 PMC6169242

[ref35] Cook E.; Griffiths J. A.; Koutalonis M.; Gent C.; Pani S.; Horrocks J.; George L.; Hardwick S.; Speller R. In Illicit Drug Detection Using Energy Dispersive X-Ray Diffraction, Non-Intrusive Inspection Technologies II, Proceedings Of Spie - The International Society For Optical Engineering; SPIE, 2009; p 731001.

[ref36] Shaw L.; Dennany L. Applications Of Electrochemical Sensors: Forensic Drug Analysis. Curr. Opin. Electrochem. 2017, 3, 23–28. 10.1016/J.Coelec.2017.05.001.

[ref37] Beduk T.; Beduk D.; de Oliveira Filho J. I.; de Oliveira Filho J. I.; Zihnioglu F.; Cicek C.; Sertoz R.; Arda B.; Goksel T.; Turhan K.; Salama K. N.; Timur S. Rapid Point-Of-Care Covid-19 Diagnosis With A Gold-Nanoarchitecture-Assisted Laser-Scribed Graphene Biosensor. Anal. Chem. 2021, 93, 8585–8594. 10.1021/Acs.Analchem.1c01444. 34081452

[ref38] aLundahl L. H.; Cannoy C. Covid-19 And Substance Use In Adolescents. Pediatr. Clin. North Am. 2021, 68, 977–990. 10.1016/J.Pcl.2021.05.005. 34538307 PMC8445753

[ref39] Vivaldi F. M.; Dallinger A.; Bonini A.; Poma N.; Sembranti L.; Biagini D.; Salvo P.; Greco F.; Di Francesco F. Three-Dimensional (3d) Laser-Induced Graphene: Structure, Properties, And Application To Chemical Sensing. ACS Appl. Mater. Interfaces 2021, 13, 30245–30260. 10.1021/Acsami.1c05614. 34167302 PMC8289247

[ref40] Joshi P.; Shukla S.; Gupta S.; Riley P. R.; Narayan J.; Narayan R. Excimer Laser Patterned Holey Graphene Oxide Films For Nonenzymatic Electrochemical Sensing. ACS Appl. Mater. Interfaces 2022, 14, 37149–37160. 10.1021/Acsami.2c09096. 35930801

[ref41] Nufer S.; Lynch P. J.; Large M. J.; Ogilvie S. P.; Salvage J. P.; Pelaez-Fernandez M.; Waters T.; Jurewicz I.; Muñoz E.; Arenal R.; et al. Laser-Deposited Carbon Aerogel Derived From Graphene Oxide Enables No2-Selective Parts-Per-Billion Sensing. ACS Appl. Mater. Interfaces 2020, 12, 39541–39548. 10.1021/Acsami.0c09112. 32697564

[ref42] Meng L.; Turner A. P. F.; Mak W. C. Conducting Polymer-Reinforced Laser-Irradiated Graphene As A Heterostructured 3d Transducer For Flexible Skin Patch Biosensors. ACS Appl. Mater. Interfaces 2021, 13, 54456–54465. 10.1021/Acsami.1c13164. 34726900 PMC8603349

[ref43] Ghanam A.; Lahcen A. A.; Beduk T.; Alshareef H.; Amine A.; Salama K. N. Laser Scribed Graphene: A Novel Platform For Highly Sensitive Detection Of Eletroactive Biomolecules. Biosens. Bioelectron. 2020, 168, 112509 10.1016/J.Bios.2020.112509. 32877778

[ref44] Rauf S.; Lahcen A. A.; Aljedaibi A.; Beduk T.; de Oliveira Filho J. I.; Salama K. N. Gold Nanostructured Laser-Scribed Graphene: A New Electrochemical Biosensing Platform For Potential Point-Of-Care Testing Of Disease Biomarkers. Biosens. Bioelectron. 2021, 180, 113116 10.1016/J.Bios.2021.113116. 33662847

[ref45] Li Q.; Wu T.; Zhao W.; Ji J.; Wang G. Laser-Induced Corrugated Graphene Films For Integrated Multimodal Sensors. ACS Appl. Mater. Interfaces 2021, 13, 37433–37444. 10.1021/Acsami.1c12686. 34324306

[ref46] Beduk D.; de Oliveira Filho J. I.; Beduk T.; Harmanci D.; Zihnioglu F.; Cicek C.; Sertoz R.; Arda B.; Goksel T.; Turhan K.; et al. ’All In One′ Sars-Cov-2 Variant Recognition Platform: Machine Learning-Enabled Point Of Care Diagnostics. Biosens Bioelectron.: X 2022, 10, 100105 10.1016/J.Biosx.2022.100105FromNlm. 35036904 PMC8743487

[ref47] Beduk T.; Lahcen A. A.; Tashkandi N.; Salama K. N. One-Step Electrosynthesized Molecularly Imprinted Polymer On Laser Scribed Graphene Bisphenol A Sensor. Sens. Actuators, B 2020, 314, 128026 10.1016/J.Snb.2020.128026.

[ref48] Eissa S.; Almthen R. A.; Zourob M. Disposable Electrochemical Immunosensor Array For The Multiplexed Detection Of The Drug Metabolites Morphine, Tetrahydrocannabinol And Benzoylecgonine. Microchim. Acta 2019, 186, 523 10.1007/S00604-019-3646-8. 31292788

[ref49] Mcgeehan J.; Dennany L. Electrochemiluminescent Detection Of Methamphetamine And Amphetamine. Forensic Sci. Int. 2016, 264, 1–6. 10.1016/J.Forsciint.2016.02.048. 26978790

[ref50] Mahmoudi T.; De La Guardia M.; Shirdel B.; Mokhtarzadeh A.; Baradaran B. Recent Advancements In Structural Improvements Of Lateral Flow Assays Towards Point-Of-Care Testing. TrAC, Trends Anal. Chem. 2019, 116, 13–30. 10.1016/J.Trac.2019.04.016.

[ref51] Ahmad R.; Surya S. G.; Sales J. B.; Mkaouar H.; Catunda S. Y. C.; Belfort D. R.; Lei Y.; Wang Z. L.; Baeumner A.; Wolfbeis O. S. In Kaustat: A Wireless, Wearable, Open-Source Potentiostat For Electrochemical Measurements, 2019 IEEE Sensors; IEEE, 2019; pp 1–4.

