# Supplementary material for: Smart Multiplex Point-of-Care Platform for Simultaneous Drug Monitoring
Source: ACS Appl Mater Interfaces. 2023 Jul 27;15(31):37247–58. doi: 10.1021/acsami.3c06461 (PMC10416146; doi:10.1021/acsami.3c06461)
Supplement: Supplementary file 1 — am3c06461_si_001.pdf [file am3c06461_si_001.pdf]

Supporting Information

TO

A Smart Multiplex Point-of-Care Platform For Simultaneous Drug Monitoring

Duygu Beduk,[a] Tutku Beduk,[b,c] José Ilton de Oliveira Filho,[c] Abdellatif Ait Lahcen,[d] Ebru Aldemir,[e] Emine Guler Celik,[f] Khaled Nabil Salama,[c]* Suna Timur,[a,g]*

[a]Central Research Test and Analysis Laboratory Application and Research Center, Ege University, 35100 Bornova, Izmir, Turkey

[b]Silicon Austria Labs (SAL) GmbH, Europastraße 12, 9500, Villach, Austria

[c]Sensors Lab, Advanced Membranes and Porous Materials Center, Computer, Electrical, and Mathematical Science and Engineering Division, King Abdullah University of Science and Technology (KAUST), Thuwal 23955-6900 Saudi Arabia

[d]Dalio Institute for Cardiovascular Imaging, Department of Radiology, Weill Cornell Medicine, New York, NY 10021, USA

[e]Department of Psychiatry, Faculty of Medicine, Izmir Tınaztepe University, 35400, Buca, Izmir, Turkey

[f]Department of Bioengineering, Faculty of Engineering, Ege University, 35100, Bornova, Izmir, Turkey

[g]Department of Biochemistry, Faculty of Science, Ege University, 35100 Bornova, Izmir, Turkey

*Corresponding authors: suna.timur@ege.edu.tr, khaled.salama@kaust.edu.sa

1.Multiplex LSG fabrication

The multiplex graphene sensor was designed to provide simultaneous detection of multiple stimulants in one biological media complex. The design has one reference electrode, one counter electrode, and three working electrodes made by a one-step laser scribing process directly on PI substrate under an inert atmosphere without the need for a mask. Necessary atomic rearrangement of imide and aromatic reporting units of PI takes place at high temperatures during scribing, leading to the formation of sp^2 bonded carbon atoms. [1] The best multilayer graphene form was achieved with the parameters of 3.2 W power, 2.8 cm/s speed, 1000 pulses per inch, and 2.5 mm z distance. To eliminate the excess oxygen species from the surface during the production of graphene, nitrogen gas flow was applied in during the process.

2.Electrochemical Measurements.

All electrochemical optimization measurements were performed repeatedly at room temperature by using 0.1 M KCl containing 5 mM $[Fe(CN)_{6]}^{3-/4-}$ as a redox probe. Cyclic voltammetry was used to clean the working electrode surface and check the stability of multiplex LSG electrodes. After antibody immobilization onto the surface and binding analyte, differential pulse voltammetry (DPV) were used to identify the electrodes and analyze sensor response. The changes in oxidation current values are changed correspondingly to the bonding of immobilized drug antibodies and the different concentrations of drug analytes. All electrochemical measurements were performed 6 times at pH 7.4. The ultrapure 18.2 MΩ cm Milli-Q water was used for preparing the aqueous solutions. The scan rate for CV and DPV was 50 mV/s, and the potential range was between -0.60 V and + 0.40 V. All electrochemical performance test measurements were performed by KAUSTat in 5.0 mM $[Fe(CN)_6]^{3-/4-}$ containing 0.1 M PBS and 0.1 M KCl. Following the interference measurements, the r*elative peak height reduction* was calculated by taking the minimum oxidation response as the reference point in a set of measurements. The same representation was used fo the real sample tests and the effect of lyrica. The calculation was calculated from the equation below:

$$\text{Relative peak height } reduction\ \% = \frac{\Delta I_{interference}}{minimum\ \Delta I_{interference}} \times 100$$

$(\Delta I)_{interference}$: The oxidation current response difference between the antibody and analyte

Minimum $(\Delta I)_{interference}$: The minimum oxidation current response difference between the antibody and analyte obtained from a set of measurements.

The multiplex hand-held potentiostat device was initially developed to perform electrochemical measurements for single analyte measurement.[2] To date, various versions have been developed for various point of care applications such as COVID-19 diagnosis, and early breast cancer detection. [3] In addition, we have demonstrated the use for on-site environmental use of KAUSTat by creating a sensing platform for Bisphenol A as a pollutant in water supplies. [4] This customized potentiostat operates wirelessly through a smartphone application developed by us, having multiple operations, data visualization, and communication settings. For this work, the first multiplex version of KAUSTat has been developed with eight simultaneous working electrode channels. Device circuitry and the smartphone application interface are given in Figure S1.

The potentiostat device used in this work is based on the ESP32 microcontroller (MCU) from Espressif Systems. This microcontroller is a dual-core system with two Harvard Architecture Xtensa LX6 CPUs. The MCU controls the analog-front end (AFE) circuitry using a serial peripheral interface (SPI) and measures the current from the trans-impedance amplifier (TIA) using its internal ADC. The AFE is composed of an MCP4728 chip from Microchip, providing four Digital-to-Analog (DAC) outputs, four operational amplifiers in buffers configuration in all output stages, and three operational amplifiers in TIA configuration. The potentiostat is controlled by Bluetooth, through Generic Attribute Profile (GATT) protocol. A custom-made mobile application was developed to send commands and receive data from the sensor.

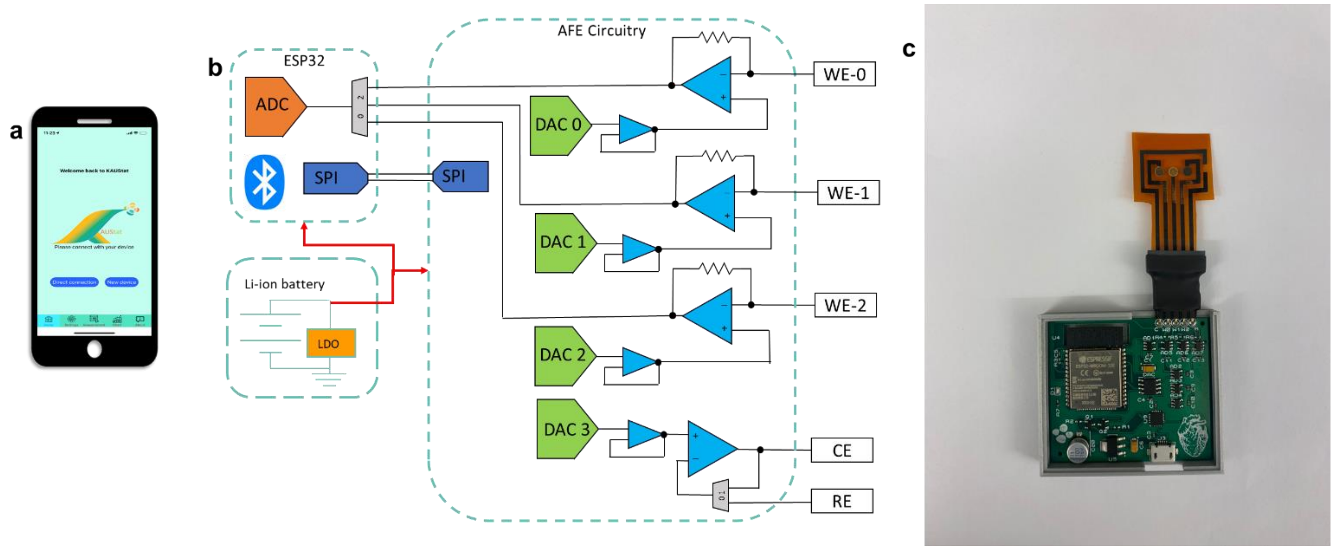

Figure S1. a) Smartphone application, **b)** lightweight potentiostat circuitry **c)** Photo of the multiplex sensor and the KAUSTat.

3.Characterizations

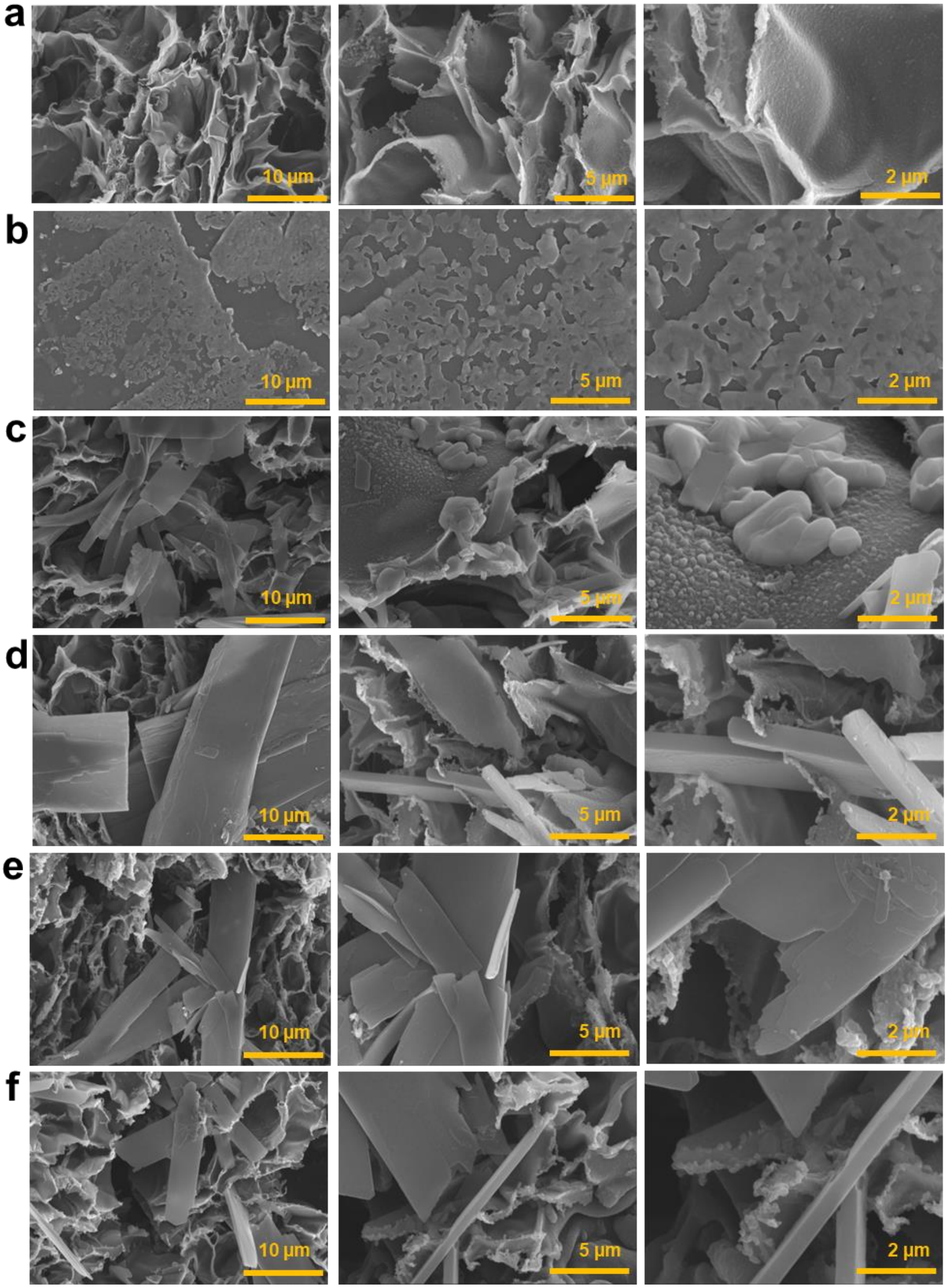

Figure S2. Scanning Electron Microscopy (SEM) images of **(a)** Bare LSG, **(b)** PBA/LSG, **(c)** EDC/NHS/PBA/LSG Sensor, **(d)** AMP Antibody/EDC/NHS/PBA/LSG Sensor, **(e)** BZD

Antibody/EDC/NHS/PBA/LSG Sensor, **(f)** COC Antibody/EDC/NHS/PBA/LSG Sensor in different magnifications.

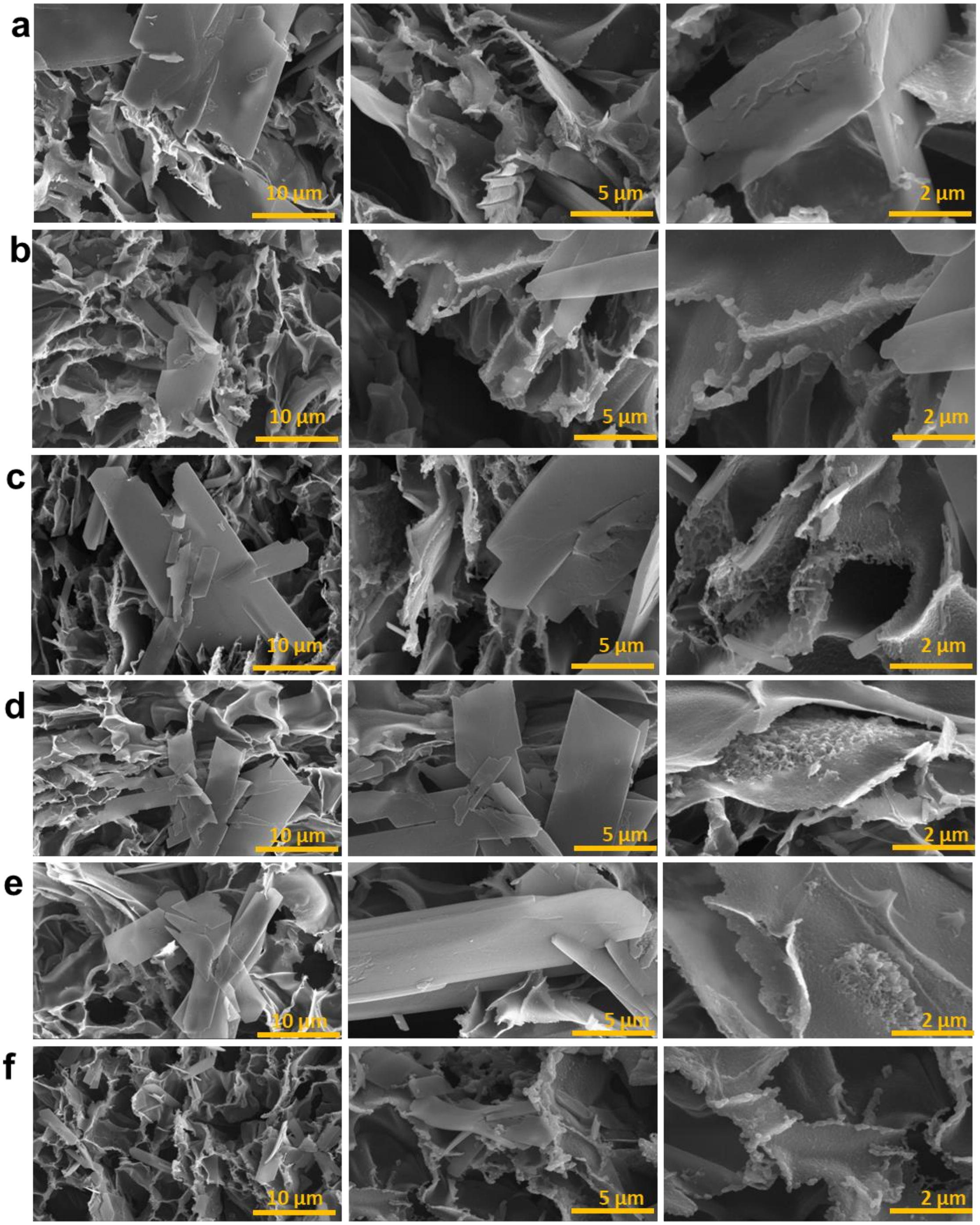

Figure S3. Scanning Electron Microscopy (SEM) images of **(a)** BSA/COC Antibody/ EDC/NHS/PBA/LSG Sensor, **(b)** BSA/BZD Antibody/EDC/NHS/PBA/LSG Sensor, **(c)** BSA/AMP Antibody/ EDC/NHS/PBA/LSG Sensor, **(d)** COC/BSA/COC Antibody/ EDC/NHS/PBA/LSG Sensor,

e)BZD/BSA/BZD Antibody/ EDC/NHS/PBA/LSG Sensor, **(f)** AMP/BSA/AMP Antibody/ EDC/NHS/PBA/LSG Sensor in different magnifications

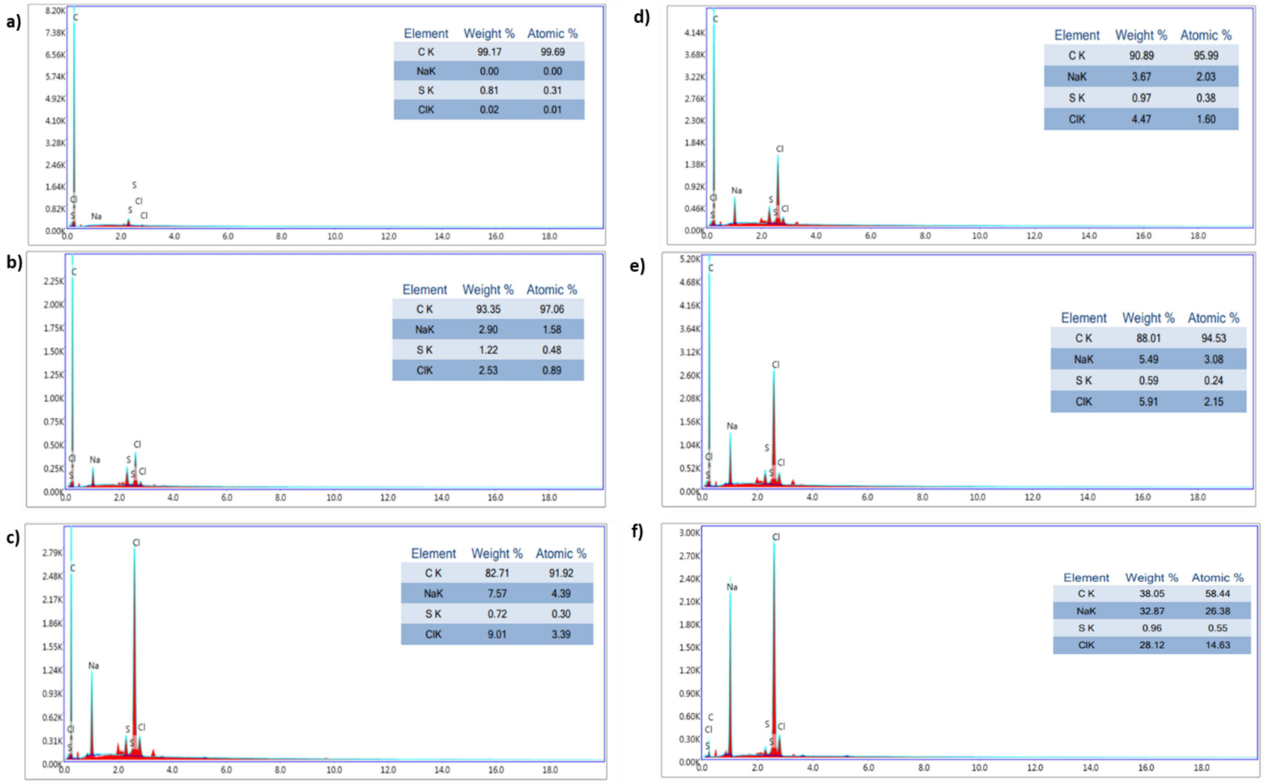

Figure S4. Energy-dispersive X-ray (EDX) spectroscopy **(a)** LSG, **(b)** PBA/LSG **(c)** EDC/NHS/PBA/LSG (d) Antibody/EDC/NHS/PBA/LSG, **(e)** BSA/Antibody/EDC/NHS/PBA/LSG, **(f)** Antigen/BSA/ Antibody/ EDC/NHS/PBA/LSG Sensor.

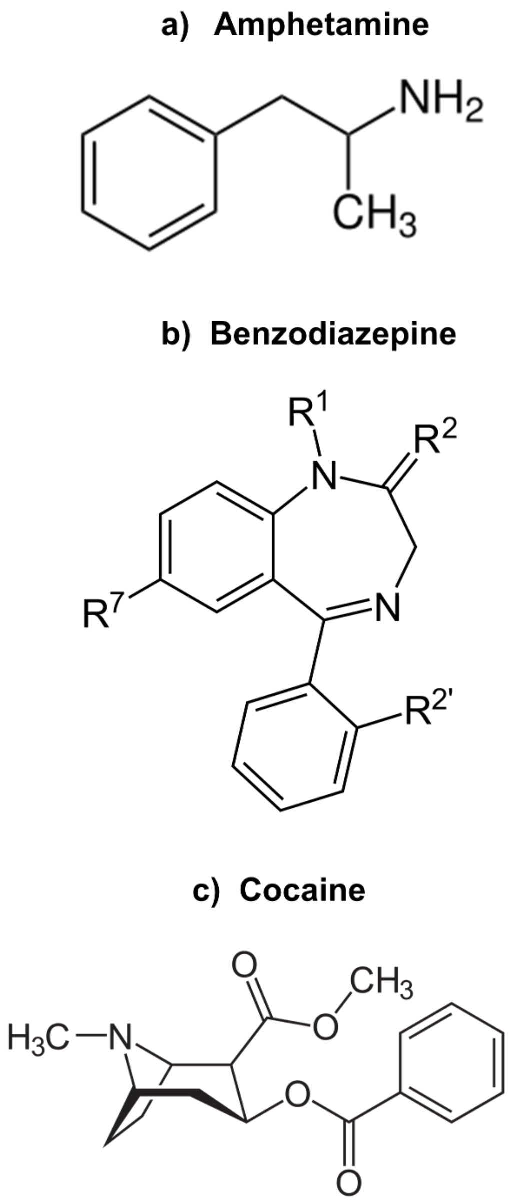

Figure S5. Chemical structures of a) Amphetamine, b) Benzodiazepine, and c) Cocaine.

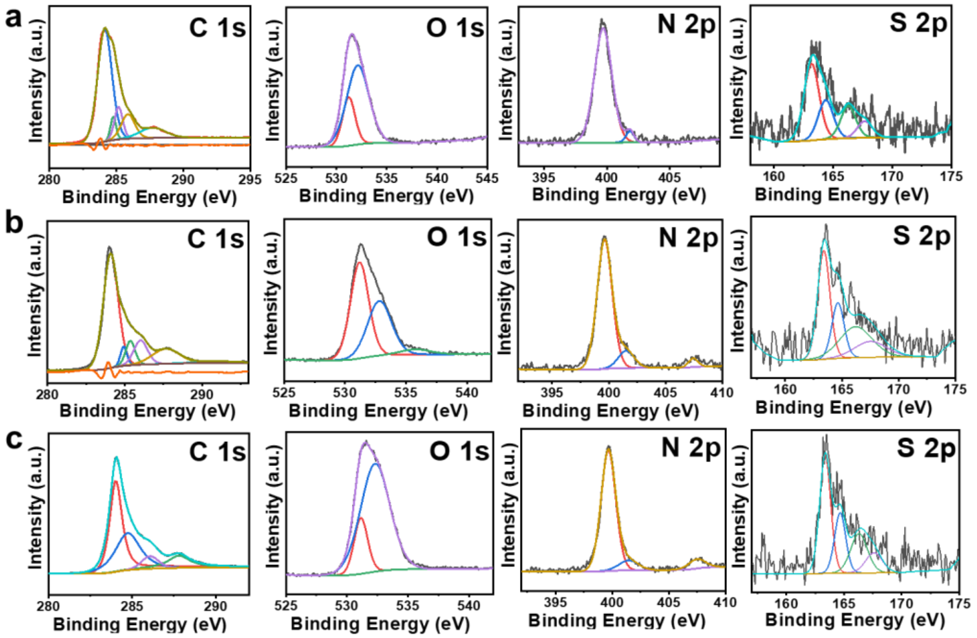

Name	Atomic %
S 2p	0.21
Cl 2p	0.68
C 1s	72.53
N 1s	5.61
O 1s	9.62
Na 1s	0.5

Name	Atomic %
S 2p	0.33
Cl 2p	0.64
C 1s	66.72
N 1s	6.29
O 1s	13.92
Na 1s	0.92

Name	Atomic %
S 2p	0.35
Cl 2p	0.8
C 1s	70.54
N 1s	5.83
O1s	10.48
Na1s	1.08

Figure S6. XPS High resolution spectra of **(a)** Bare LSG, **(b)** PBA/LSG, **(c)** EDC/NHS/PBA/LSG Sensor.

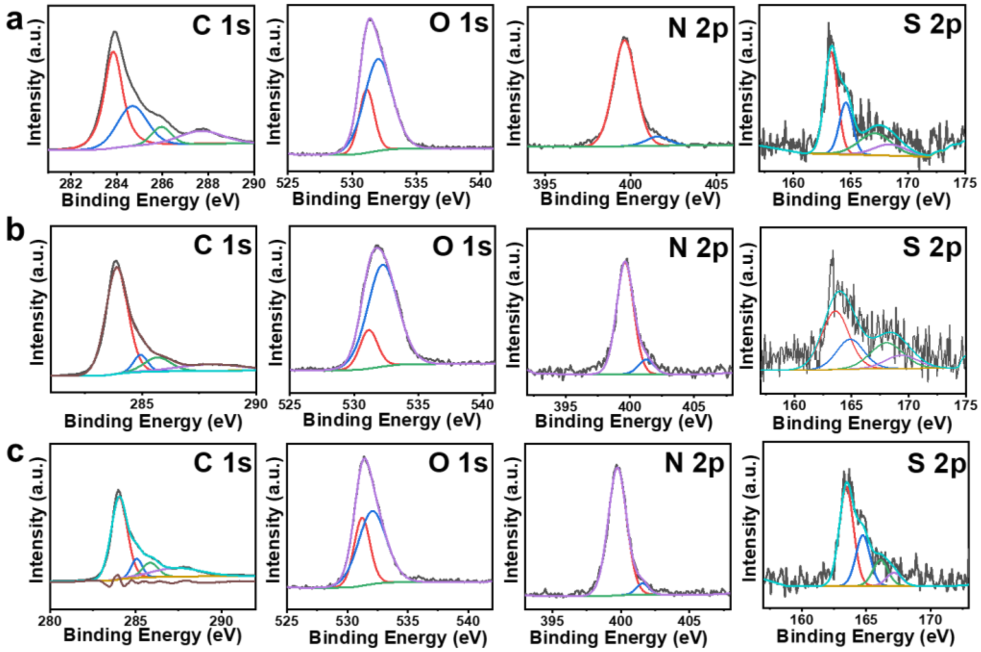

Name	Atomic %
S 2p	0.23
Cl 2p	0.56
C 1s	68.53
N 1s	7.01
O1s	11.85
Na 1s	0.84

Name	Atomic %
Cl 2p	0.43
S 2s	0.31
C 1s	74.55
N 1s	3.52
O1s	9.2
Na 1s	0.82

Name	Atomic %
S 2p	0.35
Cl 2p	0.7
C 1s	70.71
N 1s	5.65
O1s	10.6
Na 1s	0.96

Figure S7. XPS High resolution spectra of **(a)** BSA/COC Antibody/ EDC/NHS/PBA/LSG Sensor, **(b)** BSA/BZD Antibody/ EDC/NHS/PBA/LSG Sensor, **(c)** BSA/AMP Antibody/ EDC/NHS/PBA/LSG Sensor.

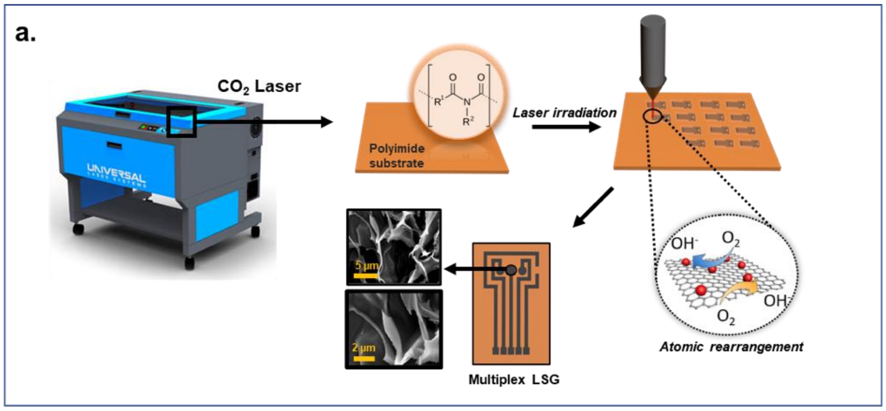

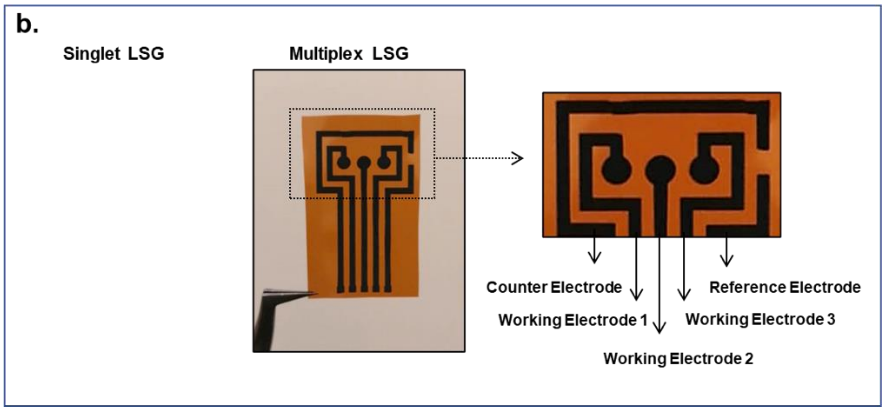

Figure S8. **(a)** Fabrication procedure of LSG sensors. **(b)** Actual image of singlet LSG and Multiplex LSG sensors, including counter, reference and working electrodes.

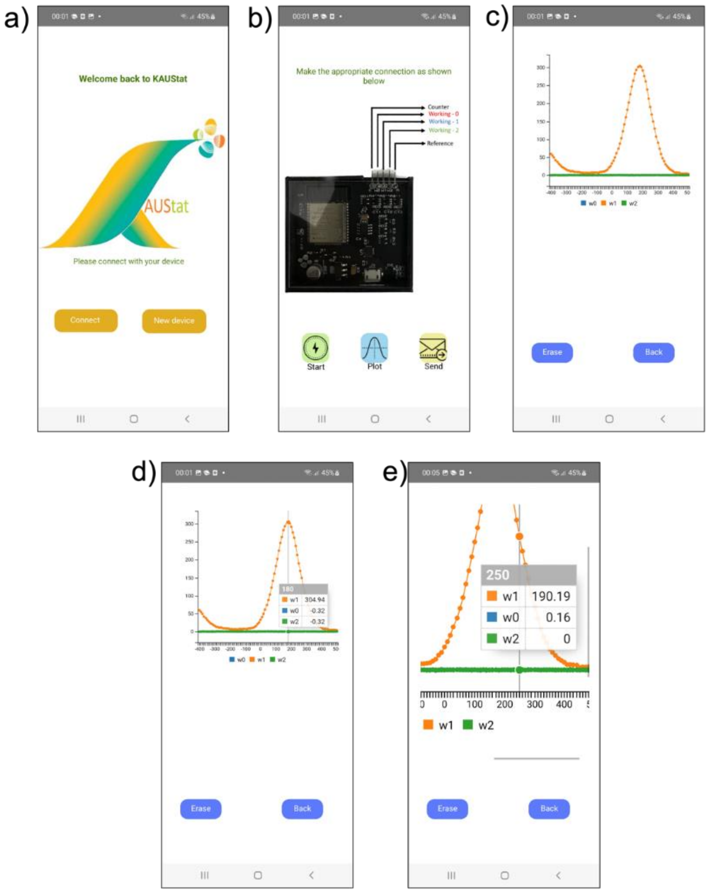

Figure S9. Screenshots of KAUSTat app control with **(a)** connection screen, **(b)** control screen, and **(c)** data visualization screen **(d)** data visualization screen with measured W0,W1,W2 values **(e)** zoomed data screen. The peak current or other features of the curve are not calculated in the app, the user can only zoom the graph or select the values of the curve. The image created can be saved by copying the image and the data is sent by email in .txt file format. The mobile application is used only for early visualization, the data can be further pos-processed and visualized in third-party software.

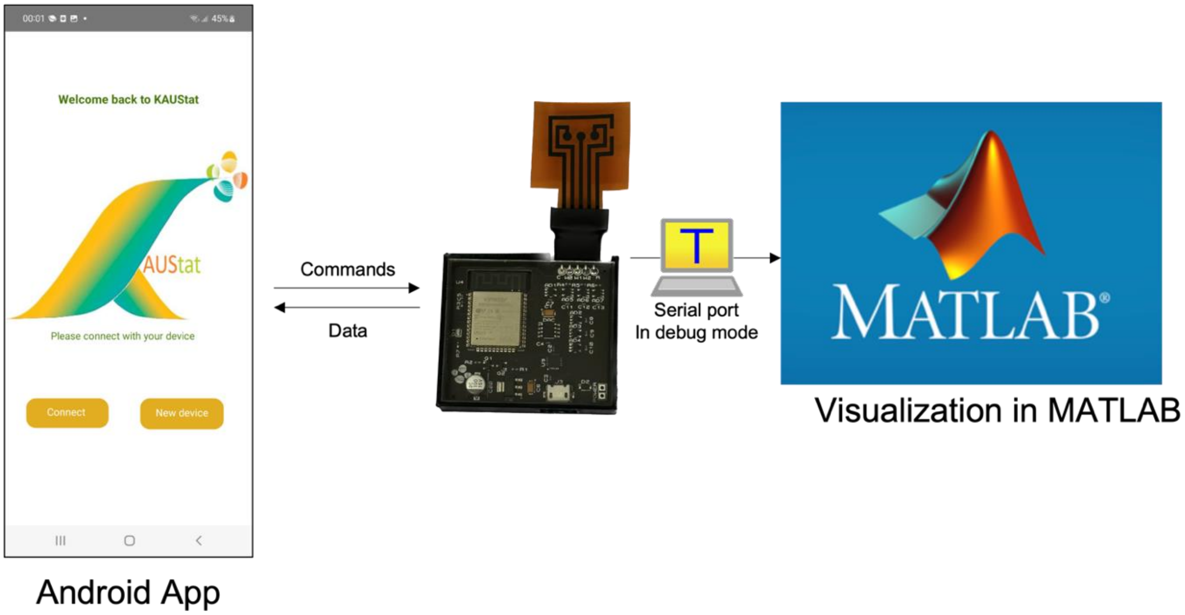

Figure S10. In debug mode, the mobile application controls the device, and the data is exported directly into Matlab for processing and visualization. The app controls the device using the GATT protocol and the data is transferred to the computer using the UART serial protocol.

Table S1. Summary of reported electrochemical biosensing systems for COC, AMP, and BZD

Target Analyte	Detection Platform	Electrode Type	Media	LOD	Ref
Cocaine	Polymer-based Biosensor	PPP-CD-*g*-PEG/GCE	Spiked Synthetic Urine Samples	28.62 nM	5
Cocaine	Immunosensor	CoNTA-Ab/SPE	Spiked Synthetic Serum, Sweat, Urine,Saliva	3.6 ng/mL	6
Cocaine	Aptasensor	ITO Electrode	Spiked Human Blood Serum	0.0335 ng/mL	7
Cocaine	Polymer-based Biosensor	Poly(PABA)/ GSPE	Street Samples	50 μM	8
Cocaine	Aptasensor	SPGE	Spiked Rat Blood Serum	273 pM	9
Cocaine	Apstasensor	AuNPs/rGO/SPCE	Synthetic Standards	1 nM	10
Cocaine	Aptasensor	SPGE	Spiked Rat Blood Serum	136 pM	11
Cocaine	Apstasensor	AgNPs/MWCNTs-IL-Chit/GCE	Spiked Human Blood Serum	150 pM	12
Cocaine	Hydrogel-based Sensor	SPE	Street Samples	2 μM	13

Cocaine	Aptasensor	NPGE	Synthetic Standards	21 nM	14
Cocaine	Aptasensor	Au/Cy3S/MCE/GE	Spiked Human Blood Serum	100 nM	15
Cocaine	Aptasensor	Cell GCE	Spiked Human Blood Serum	30 nmol/L	16
Cocaine	Aptasensor	CDs-ITO	Spiked Human Serum and Urine	0.26 pM	17
Cocaine	Aptasensor	AuNP/GCE	Spiked Human Blood Serum	0.5 pM	18
Cocaine	Electrochemical profile-based Sensor	GSPE	Street Samples	3 μM	19
Cocaine	Aptasensor	AuNP-SPE	Serum Samples	15 pM	20
Amphetamine	Electrochemical profile-based Sensor	Paraformaldehyde-coated GSPE	Seized samples	0.3 mM	21
Amphetamine	Microsensor	Ion-selective Microelectrode	Synthetic Standards	12 μM	22
Amphetamine	Microsensor	Platinum Microelectrode	Synthetic Standards	40 μM	23
Amphetamine	Derivatized sensor	GSPE	Seized samples	22.2 μM	24
Amphetamine	Aptasensor	AuNFs	Spiked Human Urine	0.51 nM	25
Benzodiazepine (Flunitrazepam)	Electrochemical profile-based Sensor	TiO2@CuO-N-rGO/poly (L-Cys)/GCE	Spiked Plasma Samples	0.3 nM	26
Benzodiazepine (Flunitrazepam)	Electrochemical profile-based Sensor	$MnFe_2O_4$/AuNPs/CPE	Spiked Human Plasma	0.33 μM	27
Benzodiazepine (Oxazepam)	Electrochemical profile-based Sensor	Ag-Pt/**GRs**/GCE	Spiked Urine and Human Blood Serum	42 Nm	28
Benzodiazepine (Nitrazepam)	Electrochemical profile-based Sensor	AuNPs/rGO/GCE	Spiked Human Blood Serum	0.166 μM	29
Benzodiazepine (Clonazepam)	Electrochemical profile-based Sensor	AgNPs/MWCNTs/GCE	Spiked Human Blood Serum	6.0 nM	30
Benzodiazepine (Clonazepam)	Electrochemical profile-based Sensor	SFs – IL/GCE	Spiked Serum and Urine Samples	66 nM	31
Benzodiazepine (Clonazepam)	Electrochemical profile-based Sensor	MWCNTs/ZnO-NPs/CPE	Spiked Human Urine	0.173 μg/mL	32
Benzodiazepine (Diazepam)	Electrochemical profile-based Sensor	C60-CNTs/IL/GCE	Spiked Human Blood Serum	87 ± 2 nM	33
Benzodiazepines (Clonazepam, Diazepam, Alprazolam, Chlordiazepoxide, Oxazepam)	Electrochemical profile-based Sensor	P(DA-FA)/GCE	Spiked Human Plasma	-	34
Benzodiazepines	Electrochemical profile-based Sensor	Ag/N-GQD/GE	Spiked Human Plasma	-	35

(Alprazolam, Chlordiazepoxide bis, Diazepam, Oxazepam and Clonazepam)					
Amphetamine<br>Methamphetamine	Electrochemiluminescence Sensor	GCE	Street Samples	-	36
Morphine<br>THC<br>Benzoylecgonine	Immunosensor	Multiplex Au-DEP	Spiked Urine	LOD of MOR:1.2 pg/mL<br>LOD of THC: 7.0 pg/mL<br>LOD of BZC:8.0 pg/mL	37
**Amphetamine**<br>**Benzodiazepine**<br>**Cocaine**	**Immunosensor**	**Multiplex LSG**	**Spiked Human Saliva**	**LOD of AMP: 4.27 ng/ml**<br>**LOD of BZD: 9.7 ng/ml**<br>**LOD of COC: 9.01 ng/ml**	**This Work**

Abbreviations: GE: Gold Electrode; GCE: Glassy Carbon Electrode; SPE: Screen Printed Electrode; SPGE: Screen Printed Gold Electrode; SPCE: Screen Printed Carbon Electrode; GSPE: Graphene Screen Printed Carbon Electrode; PPP-CD-g-PEG: Poly(p-phenylene)-Cyclodextrin-Poly(ethylene glycol); CoNTA: Cobalt Oxide Nitrilotriacetate; Ab: Antibody; ITO: Indium Tin Oxide; Poly(PABA): Poly(Para-Aminobenzoic Acid); AuNPs: Gold Nanopatricles; rGO: Reduced Graphene Oxide; SFs: Silver Fibers; MWCNTs: Multi-Walled Carbon Nanotubes; ZnO: Zinc Oxide; Chit: Chitosan; NPGE: Nanoporous Gold Electrode; Cy3S: Aptamer fragment; MCE: Mercaptoethanol; CDs: Carbon Dots; AuNFs: Gold Nanoflowers; TiO_2: Titanium Oxide; L-Cys: L-cysteine; CuO: Copper(II) oxide; N-rGO: Nitrogen doped reduced graphene oxide; $MnFe_2O_4$: Manganese Ferrite Nanoparticles; CPE: Carbon Paste Electrode; Ag: Silver; Pt: Platinum; GRs: Graphene Nanpsheets; P(DA-FA): Poly dopamine-poly folic acid; Ag/N-GQD: Silver Nanoparticle-nitrogen doped Graphene Quantum Dots; C60: Fullerene; CNTs: Carbon Nanotubes; IL: Ionic Liquid; DEP: Disposable Electrical Printed LSG: Laser Scribed Graphene

References

(1) Lin, J.; Peng, Z.; Liu, Y.; Ruiz-Zepeda, F.; Ye, R.; Samuel, E. L. G.; Yacaman, M. J.; Yakobson, B. I.; Tour, J. M. Laser-induced porous graphene films from commercial polymers. *Nature Communications* **2014**, *5* (1), 5714. DOI: 10.1038/ncomms6714.

(2) Ahmad, R.; Surya, S. G.; Sales, J. B.; Mkaouar, H.; Catunda, S. Y. C.; Belfort, D. R.; Lei, Y.; Wang, Z. L.; Baeumner, A.; Wolfbeis, O. S.; et al. KAUSTat: A Wireless, Wearable, Open-Source Potentiostat for Electrochemical Measurements. In *2019 IEEE SENSORS*, 27-30 Oct. 2019, 2019; pp 1-4. DOI: 10.1109/SENSORS43011.2019.8956815.

(3) Rauf, S.; Lahcen, A. A.; Aljedaibi, A.; Beduk, T.; Ilton de Oliveira Filho, J.; Salama, K. N. Gold nanostructured laser-scribed graphene: A new electrochemical biosensing platform for potential point-of-care testing of disease biomarkers. *Biosensors and Bioelectronics* **2021**, *180*, 113116. DOI: https://doi.org/10.1016/j.bios.2021.113116. Beduk, T.; Beduk, D.; de Oliveira Filho, J. I.; Zihnioglu, F.; Cicek, C.; Sertoz, R.; Arda, B.; Goksel, T.; Turhan, K.; Salama, K. N.; et al. Rapid Point-of-Care COVID-19 Diagnosis with a Gold-Nanoarchitecture-Assisted Laser-Scribed Graphene Biosensor. *Analytical Chemistry* **2021**, *93* (24), 8585-8594. DOI: 10.1021/acs.analchem.1c01444.

(4) Beduk, T.; Gomes, M.; De Oliveira Filho, J. I.; Shetty, S. S.; Khushaim, W.; Garcia-Ramirez, R.; Durmus, C.; Lahcen, A. A.; Salama, K. N. J. F. i. c. A Portable Molecularly Imprinted Sensor for On-Site and Wireless Environmental Bisphenol A Monitoring. **2022**, *10*.

(5) Yilmaz Sengel, T.; Guler, E.; Arslan, M.; Gumus, Z. P.; Sanli, S.; Aldemir, E.; Akbulut, H.; Odaci Demirkol, D.; Coskunol, H.; Timur, S.; et al. "Biomimetic-electrochemical-sensory-platform" for biomolecule free cocaine testing. *Materials Science and Engineering: C* **2018**, *90*, 211-218. DOI: https://doi.org/10.1016/j.msec.2018.04.043.

(6) Sanli, S.; Moulahoum, H.; Ugurlu, O.; Ghorbanizamani, F.; Gumus, Z. P.; Evran, S.; Coskunol, H.; Timur, S. Screen printed electrode-based biosensor functionalized with magnetic cobalt/single-chain

antibody fragments for cocaine biosensing in different matrices. *Talanta* **2020**, *217*, 121111. DOI: https://doi.org/10.1016/j.talanta.2020.121111.
(7) Wang, J.; Liu, J.; Wang, M.; Qiu, Y.; Kong, J.; Zhang, X. A host guest interaction enhanced polymerization amplification for electrochemical detection of cocaine. *Analytica Chimica Acta* **2021**, *1184*, 339041. DOI: https://doi.org/10.1016/j.aca.2021.339041.
(8) Florea, A.; Cowen, T.; Piletsky, S.; De Wael, K. Polymer platforms for selective detection of cocaine in street samples adulterated with levamisole. *Talanta* **2018**, *186*, 362-367. DOI: https://doi.org/10.1016/j.talanta.2018.04.061.
(9) Abnous, K.; Danesh, N. M.; Ramezani, M.; Taghdisi, S. M.; Emrani, A. S. A novel electrochemical aptasensor based on H-shape structure of aptamer-complimentary strands conjugate for ultrasensitive detection of cocaine. *Sensors and Actuators B: Chemical* **2016**, *224*, 351-355. DOI: https://doi.org/10.1016/j.snb.2015.10.039.
(10) Jiang, B.; Wang, M.; Chen, Y.; Xie, J.; Xiang, Y. Highly sensitive electrochemical detection of cocaine on graphene/AuNP modified electrode via catalytic redox-recycling amplification. *Biosensors and Bioelectronics* **2012**, *32* (1), 305-308. DOI: https://doi.org/10.1016/j.bios.2011.12.010.
(11) Taghdisi, S. M.; Danesh, N. M.; Emrani, A. S.; Ramezani, M.; Abnous, K. A novel electrochemical aptasensor based on single-walled carbon nanotubes, gold electrode and complimentary strand of aptamer for ultrasensitive detection of cocaine. *Biosensors and Bioelectronics* **2015**, *73*, 245-250. DOI: https://doi.org/10.1016/j.bios.2015.05.065.
(12) Roushani, M.; Shahdost-fard, F. A novel ultrasensitive aptasensor based on silver nanoparticles measured via enhanced voltammetric response of electrochemical reduction of riboflavin as redox probe for cocaine detection. *Sensors and Actuators B: Chemical* **2015**, *207*, 764-771. DOI: https://doi.org/10.1016/j.snb.2014.10.131.
(13) de Jong, M.; Sleegers, N.; Kim, J.; Van Durme, F.; Samyn, N.; Wang, J.; De Wael, K. Electrochemical fingerprint of street samples for fast on-site screening of cocaine in seized drug powders. *Chemical Science* **2016**, *7* (3), 2364-2370, 10.1039/C5SC04309C. DOI: 10.1039/C5SC04309C.
(14) Tavakkoli, N.; Soltani, N.; Mohammadi, F. A nanoporous gold-based electrochemical aptasensor for sensitive detection of cocaine. *RSC Advances* **2019**, *9* (25), 14296-14301, 10.1039/C9RA01292C. DOI: 10.1039/C9RA01292C.
(15) Zhang, D.-W.; Zhang, F.-T.; Cui, Y.-R.; Deng, Q.-P.; Krause, S.; Zhou, Y.-L.; Zhang, X.-X. A label-free aptasensor for the sensitive and specific detection of cocaine using supramolecular aptamer fragments/target complex by electrochemical impedance spectroscopy. *Talanta* **2012**, *92*, 65-71. DOI: https://doi.org/10.1016/j.talanta.2012.01.049.
(16) Hua, M.; Li, P.; Li, L.; Huang, L.; Zhao, X.; Feng, Y.; Yang, Y. Quantum dots as immobilized substrate for electrochemical detection of cocaine based on conformational switching of aptamer. *Journal of Electroanalytical Chemistry* **2011**, *662* (2), 306-311. DOI: https://doi.org/10.1016/j.jelechem.2011.08.017.
(17) Azizi, S.; Gholivand, M. B.; Amiri, M.; Manouchehri, I.; Moradian, R. Carbon dots-thionine modified aptamer-based biosensor for highly sensitive cocaine detection. *Journal of Electroanalytical Chemistry* **2022**, *907*, 116062. DOI: https://doi.org/10.1016/j.jelechem.2022.116062.
(18) Roushani, M.; Shahdost-fard, F. Fabrication of an electrochemical nanoaptasensor based on AuNPs for ultrasensitive determination of cocaine in serum sample. *Materials Science and Engineering: C* **2016**, *61*, 599-607. DOI: https://doi.org/10.1016/j.msec.2016.01.002.
(19) de Jong, M.; Florea, A.; Vries, A.-M. d.; van Nuijs, A. L. N.; Covaci, A.; Van Durme, F.; Martins, J. C.; Samyn, N.; De Wael, K. Levamisole: a Common Adulterant in Cocaine Street Samples Hindering Electrochemical Detection of Cocaine. *Analytical Chemistry* **2018**, *90* (8), 5290-5297. DOI: 10.1021/acs.analchem.8b00204.
(20) Abnous, K.; Abdolabadi, A. k.; Ramezani, M.; Alibolandi, M.; Nameghi, M. A.; Zavvar, T.; Khoshbin, Z.; Lavaee, P.; Taghdisi, S. M.; Danesh, N. M. A highly sensitive electrochemical aptasensor for cocaine detection based on CRISPR-Cas12a and terminal deoxynucleotidyl transferase as signal amplifiers. *Talanta* **2022**, *241*, 123276. DOI: https://doi.org/10.1016/j.talanta.2022.123276.

(21) Schram, J.; Parrilla, M.; Slosse, A.; Van Durme, F.; Åberg, J.; Björk, K.; Bijvoets, S. M.; Sap, S.; Heerschop, M. W. J.; De Wael, K. Paraformaldehyde-coated electrochemical sensor for improved on-site detection of amphetamine in street samples. *Microchemical Journal* **2022**, *179*, 107518. DOI: https://doi.org/10.1016/j.microc.2022.107518.
(22) Gallardo-Gonzalez, J.; Saini, A.; Baraket, A.; Boudjaoui, S.; Alcácer, A.; Streklas, A.; Teixidor, F.; Zine, N.; Bausells, J.; Errachid, A. A highly selective potentiometric amphetamine microsensor based on all-solid-state membrane using a new ion-pair complex, [3,3′-Co(1,2-closo-C2B9H11)2]− [C9H13NH]+. *Sensors and Actuators B: Chemical* **2018**, *266*, 823-829. DOI: https://doi.org/10.1016/j.snb.2018.04.001.
(23) Gallardo-González, J.; Baraket, A.; Bonhomme, A.; Zine, N.; Sigaud, M.; Bausells, J.; Errachid, A. Sensitive Potentiometric Determination of Amphetamine with an All-Solid-State Micro Ion-Selective Electrode. *Analytical Letters* **2018**, *51* (3), 348-358. DOI: 10.1080/00032719.2017.1326053.
(24) Parrilla, M.; Felipe Montiel, N.; Van Durme, F.; De Wael, K. Derivatization of amphetamine to allow its electrochemical detection in illicit drug seizures. *Sensors and Actuators B: Chemical* **2021**, *337*, 129819. DOI: https://doi.org/10.1016/j.snb.2021.129819.
(25) Soni, S.; Jain, U.; Burke, D. H.; Chauhan, N. A label free, signal off electrochemical aptasensor for amphetamine detection. *Surfaces and Interfaces* **2022**, *31*, 102023. DOI: https://doi.org/10.1016/j.surfin.2022.102023.
(26) Sohouli, E.; Ghalkhani, M.; Rostami, M.; Rahimi-Nasrabadi, M.; Ahmadi, F. A noble electrochemical sensor based on TiO2@CuO-N-rGO and poly (L-cysteine) nanocomposite applicable for trace analysis of flunitrazepam. *Materials Science and Engineering: C* **2020**, *117*, 111300. DOI: https://doi.org/10.1016/j.msec.2020.111300.
(27) Asiabar, B. M.; Karimi, M. A.; Tavallali, H.; Rahimi-Nasrabadi, M. Application of MnFe2O4 and AuNPs modified CPE as a sensitive flunitrazepam electrochemical sensor. *Microchemical Journal* **2021**, *161*, 105745. DOI: https://doi.org/10.1016/j.microc.2020.105745.
(28) Khoshroo, A.; Hosseinzadeh, L.; Sobhani-Nasab, A.; Rahimi-Nasrabadi, M.; Ehrlich, H. Development of electrochemical sensor for sensitive determination of oxazepam based on silver-platinum core–shell nanoparticles supported on graphene. *Journal of Electroanalytical Chemistry* **2018**, *823*, 61-66. DOI: https://doi.org/10.1016/j.jelechem.2018.05.030.
(29) Fritea, L.; Bănică, F.; Costea, T. O.; Moldovan, L.; Iovan, C.; Cavalu, S. A gold nanoparticles - Graphene based electrochemical sensor for sensitive determination of nitrazepam. *Journal of Electroanalytical Chemistry* **2018**, *830-831*, 63-71. DOI: https://doi.org/10.1016/j.jelechem.2018.10.015.
(30) Habibi, B.; Jahanbakhshi, M. Silver nanoparticles/multi walled carbon nanotubes nanocomposite modified electrode: Voltammetric determination of clonazepam. *Electrochimica Acta* **2014**, *118*, 10-17. DOI: https://doi.org/10.1016/j.electacta.2013.11.169.
(31) Khoshroo, A.; Hosseinzadeh, L.; Sobhani-Nasab, A.; Rahimi-Nasrabadi, M.; Ahmadi, F. Silver nanofibers/ionic liquid nanocomposite based electrochemical sensor for detection of clonazepam via electrochemically amplified detection. *Microchemical Journal* **2019**, *145*, 1185-1190. DOI: https://doi.org/10.1016/j.microc.2018.12.049.
(32) Rizk, M.; Taha, E. A.; El-Alamin, M. M. A.; Hendawy, H. A. M.; Sayed, Y. M. Highly Sensitive Carbon Based Sensors Using Zinc Oxide Nanoparticles Immobilized Multiwalled Carbon Nanotubes for Simultaneous Determination of Desvenlafaxine Succinate and Clonazepam. *Journal of The Electrochemical Society* **2018**, *165* (7), H333-H341. DOI: 10.1149/2.0551807jes.
(33) Rahimi-Nasrabadi, M.; Khoshroo, A.; Mazloum-Ardakani, M. Electrochemical determination of diazepam in real samples based on fullerene-functionalized carbon nanotubes/ionic liquid nanocomposite. *Sensors and Actuators B: Chemical* **2017**, *240*, 125-131. DOI: https://doi.org/10.1016/j.snb.2016.08.144.
(34) Ashrafi, H.; Mobed, A.; Hasanzadeh, M.; Babaie, P.; Ansarin, K.; Jouyban, A. Monitoring of five benzodiazepines using a novel polymeric interface prepared by layer by layer strategy. *Microchemical Journal* **2019**, *146*, 121-125. DOI: https://doi.org/10.1016/j.microc.2018.12.064.
(35) Ashrafi, H.; Hassanpour, S.; Saadati, A.; Hasanzadeh, M.; Ansarin, K.; Ozkan, S. A.; Shadjou, N.; Jouyban, A. Sensitive detection and determination of benzodiazepines using silver nanoparticles-N-GQDs

ink modified electrode: A new platform for modern pharmaceutical analysis. *Microchemical Journal* **2019**, *145*, 1050-1057. DOI: https://doi.org/10.1016/j.microc.2018.12.017.
(36) McGeehan, J.; Dennany, L. Electrochemiluminescent detection of methamphetamine and amphetamine. *Forensic Science International* **2016**, *264*, 1-6. DOI: https://doi.org/10.1016/j.forsciint.2016.02.048.
(37) Eissa, S.; Almthen, R. A.; Zourob, M. Disposable electrochemical immunosensor array for the multiplexed detection of the drug metabolites morphine, tetrahydrocannabinol and benzoylecgonine. *Microchimica Acta* **2019**, *186* (8), 523. DOI: 10.1007/s00604-019-3646-8.
